# Dehydrodiisoeugenol inhibits colorectal cancer growth by endoplasmic reticulum stress-induced autophagic pathways

**DOI:** 10.1186/s13046-021-01915-9

**Published:** 2021-04-10

**Authors:** Changhong Li, Kui Zhang, Guangzhao Pan, Haoyan Ji, Chongyang Li, Xiaowen Wang, Xin Hu, Ruochen Liu, Longfei Deng, Yi Wang, Liqun Yang, Hongjuan Cui

**Affiliations:** 1 State Key Laboratory of Silkworm Genome Biology, College of Sericulture, Textile and Biomass sciences, Southwest University, #2, Tiansheng Rd., Beibei District, Chongqing, 400716 China; 2 Cancer Centre, Medical Research Institute, Southwest University, Chongqing, 400716 China; 3 Affiliated Hospital of Southwest University (the Ninth People’s Hospital of Chongqing), Chongqing, 400716 China

**Keywords:** Colorectal cancer, Dehydrodiisoeugenol (DEH), Autophagy inhibition, Endoplasmic reticulum (ER) stress, Anticancer agent

## Abstract

**Background:**

Dehydrodiisoeugenol (DEH), a novel lignan component extracted from nutmeg, which is the seed of *Myristica fragrans Houtt*, displays noticeable anti-inflammatory and anti-allergic effects in digestive system diseases. However, the mechanism of its anticancer activity in gastrointestinal cancer remains to be investigated.

**Methods:**

In this study, the anticancer effect of DEH on human colorectal cancer and its underlying mechanism were evaluated. Assays including MTT, EdU, Plate clone formation, Soft agar, Flow cytometry, Electron microscopy, Immunofluorescence and Western blotting were used in vitro. The CDX and PDX tumor xenograft models were used in vivo.

**Results:**

Our findings indicated that treatment with DEH arrested the cell cycle of colorectal cancer cells at the G1/S phase, leading to significant inhibition in cell growth. Moreover, DEH induced strong cellular autophagy, which could be inhibited through autophagic inhibitors, with a rction in the DEH-induced inhibition of cell growth in colorectal cancer cells. Further analysis indicated that DEH also induced endoplasmic reticulum (ER) stress and subsequently stimulated autophagy through the activation of PERK/eIF2α and IRE1α/XBP-1 s/CHOP pathways. Knockdown of PERK or IRE1α significantly decreased DEH-induced autophagy and retrieved cell viability in cells treated with DEH. Furthermore, DEH also exhibited significant anticancer activities in the CDX- and PDX-models.

**Conclusions:**

Collectively, our studies strongly suggest that DEH might be a potential anticancer agent against colorectal cancer by activating ER stress-induced inhibition of autophagy.

**Supplementary Information:**

The online version contains supplementary material available at 10.1186/s13046-021-01915-9.

## Background

Colorectal cancer (CRC), including colon cancer and rectal cancer, is presently one of the most common cancers in the world [1, 2]. It is the third-most-common health problem, as well as the fourth leading cause of mortality globally [3, 4]. Besides, colorectal cancer is the third-most common cancer in male and the second-most common cancer in females [5, 6]. Since its early symptoms are not obvious, most of the patients are diagnosed at an advanced stage usually [7, 8]. Colorectal cancer not only hurts the digestive system but also acutely impairs the lymph, liver, lung, and bone [9, 10]. Although the existing treatments such as chemotherapy, radiotherapy, and surgery are widely used, the prognosis of patients with colorectal cancer is poor [11, 12]. To aggravate the situation further, the absorption function of the intestine is reduced, particularly due to abnormalities in the intestinal mucosa [13]. This situation leads to diarrhea, abdominal pain, and other adverse symptoms that eventually affect the normal life of the patients [14]. With the development in the screening of anti-tumor drugs, more and more an increasing number of monomeric compounds from traditional Chinese medicine have been reported to play considerable roles in the treatment of a variety of tumors [15–17]. Hence, it is worthwhile to explore more effective and non-toxic drugs for the treatment of colorectal cancer [18]. This strategy could also be beneficial in improving the quality of life of the patients [19–21].

Nutmeg, the seed of an evergreen tree species *Myristica fragrans Houtt*, has been widely used as traditional Chinese medicine, whose pharmacological effects are aimed at curing discomfort of the digestive tract, abdominal distending pain, and persistent diarrhea [21, 22]. Dehydrodiisoeugenol (DEH), a monomeric compound extracted from nutmeg, has also been proven to exert several effects, such as hepato-protective, anti-thrombotic, anti-allergic, anti-oxidant, and anti-tumor [23–25]. However, the anticancer effects of DEH on colorectal cancer and the mechanism of action are still unclear. In this study, the anti-tumor activity of DEH on colorectal cancer was investigated through both the cell-derived xenograft (CDX) and patient-derived tumor xenograft (PDX) model. Our findings indicated that DEH may be a promising therapeutic application for the treatment of colorectal cancer.

## Materials and methods

### Cell culture

Normal human colon epithelial cell NCM460, human colorectal cancer cell lines (HCT 116 and SW620) and human embryonic renal cell lines (293 FT) were purchased from the American Type Culture Collection (ATCC, USA) and stored in our laboratory. The HCT 116 cells were cultured in McCoy’s 5A medium (Gibco, USA) containing 10% fetal bovine serum (FBS, Gibco) with 1% penicillin-streptomycin (P/S; Invitrogen, USA). The SW620 cells were cultured in Dulbecco’s Modified Eagle Medium (DMEM; Gibco, USA) added with 10% FBS and 1% P/S. The 293 FT cells were cultured in DMEM containing 10% FBS and 1% P/S maintained 1% G418 (Invitrogen, USA), to which 2% glutamine (Invitrogen, USA), 1% non-essential amino acids (Invitrogen, USA), and 1% sodium pyruvate (Invitrogen, USA) were added. When the cells grow up to 90–95% of the culture dishes, we passed the cells. And all the cell experiments done at the coverage reaches 40–50%. All the cells were cultured under a standard condition at 37 °C in a humidified atmosphere containing 5% CO_2_.

### Drug treatment

Dehydrodiisoeugenol (DEH, CAS No. 2680–81–1) was purchased from Chengdu Herbpurify Co., Ltd. and dissolved in Dimethyl Sulfoxide (DMSO) as 400 mM stock solutions. DEH was diluted in the corresponding culture medium or PBS for in vitro and in vivo experiments. The HCT 116 and SW620 cells were treated with DEH in different concentration (20, 40, and 60 μM, DMSO treatment as the control group) for 48 h. Unless otherwise specified note, we used 60 μM to treat cells in every single concentration experiment and used 48 h to treat cells in concentration gradient experiment. Microscopy (Olympus, Japan) was used to detect cell morphology. A hemocytometer was used to count the cell numbers. Each experiment was repeated thrice independently.

### Cell viability and proliferation assays

The viability and proliferation of CRC cells treated with DEH were determined by the 3-(4, 5-dimethylthiazol-2-yl)-2,5-diphenyltetrazolium bromide (MTT) assay (Sigma Aldrich, USA) [26]. Briefly, cells in the logarithmic phase were counted seeded (at 1.5 × 10^3^ cells/well) in 96-well plates containing 200 μL complete medium (with 10% FBS and 1% P/S added) and then attached overnight before the start of the treatment. Then, cells treated with DEH at different concentrations (10, 20, 30, 40, 50, 60, 70, and 80 μM), or culture medium containing DMSO was added to each well evenly (DMSO treatment as the control group). At the specific time points, cells were incubated with MTT (5 mg/mL or 20 μL/well) for 2.5 h. The medium containing formazan was then removed and instantly replaced by DMSO (200 μL) for dissolving the formazan complex. A microplate reader (Thermo Fisher, USA) was used to measure the absorbance at a wavelength of 560 nm. According to the OD value, the IC_50_ and cell viability were evaluated by analysis using the software GraphPad prism6. The cells were treated with DEH at 20, 40, and 60 μM (DMSO treatment as the control group) for 24, 48, and 72 h, using the above-described method. Each experiment was performed in independent triplicate.

### EdU staining

The cell’s proliferative ability was measured using Click-iT® EdU Imaging Kits (Invitrogen) according to the manufacturer’s instructions and operation manual. Briefly, 4 × 10^4^ cells were seeded in each 24-well plates, and then the adherent cells were incubated with different concentrations of DEH (20, 40, and 60 μM, DMSO treatment as the control group) for two days. Then, the cells were incubated with 10 μM EdU (Sigma Aldrich, USA) for 40 min. After fixing in 3.7% PFA in PBS for 15 min, the cells were incubated with 3% bovine serum albumin (BSA) and subsequently permeabilized in 0.5% Triton X-100. The cells were then incubated with Click-iT® reaction cocktails for 40 min at room temperature and protected from light. The nuclei were stained by DAPI for 30 min at room temperature. The fluorescence images were obtained using an inverted fluorescent microscope, and the percentage of EdU-positive stained cells was calculated from 10 random microscopic fields.

### Plate clone formation and soft agar assays

The cellular activity and proliferation were determined by the plate clone formation assay. Briefly, 800 cells were plated in 6-well plates. The adherent cells were incubated with different concentrations of DEH for 2 weeks. Then, the samples were stained with 0.1% crystal violet and captured via a digital scanner. The effect of DEH on the self-renewal and colony formation ability of the HCT 116 and SW620 cells was determined by soft agar assay [27, 28]. Briefly, 1.5 mL of basic medium containing 0.6% low-melting agarose and the above-mentioned concentrations of DEH was added to wells of culture-plates. Upon solidification, 1 mL of complete medium containing 0.3% low-melting agarose, 800 cells, and the above-indicated concentrations of DEH was added to the top of the solidified layer. The clones were observed and imaged in an inverted microscope. Finally, all the clones were stained with MTT at 37 °C for 30 min and captured by a digital scanner. The clones were visualized and quantified simultaneously.

### Flow cytometry

Cells were placed into a cell culture plate and cultured under standard conditions. The adherent cells were incubated with cell-culture medium containing different concentrations of DEH for 2 days for analysis of the cell cycle and apoptosis. For the cell cycle assay, the cells were harvested and fixed in cold 75% ethanol at 4 °C overnight. After washing with PBS to remove the residual alcohol, the cells were incubated with propidium iodide (PI; BD, USA) and RNase A (Sigma Aldrich, USA) at 37 °C for 1 h. For the apoptosis assay, cells were harvested and washed with cold PBS, and then resuspended in 100 μL binding buffer (BD, USA). Thereafter, the cells were incubated with FITC-labeled Annexin V (BD, San Jose, CA, USA) and PI at room temperature for 15 min. All the samples were analyzed by the FACS C6 (BD, USA) using Cell Quest software.

### Western blot analysis

A Western blot assay was performed to determine the level of protein expression or phosphorylation as described in our previous studies [28, 29]. Briefly, colorectal cancer cells (HCT 116 and SW620) were harvested, washed with cold PBS buffer, and then suspended in RIPA lysis buffer (Beyotime, China) containing the phosphatase inhibitor sodium fluoride (Beyotime, China) and phenylmethylsulfonyl fluoride (PMSF, Beyotime, China). The cell lysates were denatured at 100 °C for 25 min, and protein concentrations were determined using the BCA protein assay kit (Beyotime, China). A total of 45 μg protein w subjected to 10% sodium dodecyl sulfate-polyacrylamide gel electrophoresis (SDS-PAGE). The protein bands were transferred to PVDF membranes (Millipore, USA) using the Trans-Blot Turbo transfer system (Bio-Rad, USA). After blocking with 5% bovine serum albumin (BSA) in TBST buffer at room temperature for 2.5 h with gentle shaking, the PVDF membranes were incubated with antibodies against human Tubulin (1: 5000, AF1216, Beyotime, China), p21 (1:1000, #2947), CDK2 (1:1000, #2546), CDK4 (1:1000, #12790), Cyclin D1 (1:1000, #2978), Cyclin E1 (1:1000, #4129), Cyclin E2 (1: 1000; #4132), BiP (1:1000, #3177), Ero1-Lα (1:1000, #3264), PERK (1:1000; #5683), eIF2α (1: 1000, #5324), p-eIF2α (1:1000, #3398), IRE1α (1: 1000, #3294), XBP-1 s (1: 1000; #47134), CHOP (1: 1000, #2895), LC3B (1: 1000; #3868), p62 (1: 1000, #88588) and ATG7 (1: 1000; #8558) at 4 °C overnight. After washing with TBST buffer, the samples were incubated with HRP-conjugated secondary antibodies (1, 10,000, Life Technology, USA) for 2.5 h at room temperature. Finally, the protein bands were visualized through a detection-cum-analysis system (Clinx Science, China). All the above antibodies were obtained from Cell Signaling Technology, USA, unless specified otherwise.

### Transcriptome methodology and data analysis

The colorectal cancer cells were incubated with or without DEH for 2 days, after which the cells were collected and submitted to Novogene (Beijing, China) for transcriptome sequencing and analysis. The row data of RNA-sequence were analyzed and evaluated by the GESA database [30, 31]. Afterwards we filtered the data and cleared the unreliable data to cluster the differentially expressed genes. We classified these genes to determine the expression patterns of different genes under different experimental conditions to explore the transformation of the signaling pathway caused by DEH treatment. After classification, the gene difference analysis was conducted again.

### Electron microscopy

Transmission electron microscopy was used to identify the ultrastructure of the endoplasmic reticulum and autophagosome [30]. HCT 116 or SW620 cells were placed into cell culture plates. When the cell density reached 40%, the cells were incubated with or without 60 μM DEH for 2 days at 37 °C. The collected cells were fixed in 2.5% glutaraldehyde (Sigma-Aldrich, G5882) for 2 days at 4 °C. The samples were detected and analyzed by Wuhan Microscopic Biotechnology Co., Ltd. The images were processed using the software Adobe Photoshop CS6 and Adobe Illustrator CS6.

### Immunofluorescence and TUNEL assays

Cells were collected and fixed using cold methanol. After blocking with 10% BSA in PBS at 37 °C, the cells were incubated with an LC3B (D11) XP® Rabbit mAb (1: 500; CST, USA) at 4 °C overnight. The ells were then incubated with Alexa Fluor labeled secondary antibody (1: 2000; Invitrogen, USA) for 488-conjugated goat anti-rabbit and 594-conjugated goat anti-mouse at room temperature for 2 h. DAPI (1: 500) was then used to stain the nuclei, and LC3B-positive cells were captured using an Olympus FV1000 confocal fluorescence microscope [32]. The TUNEL detection is suitable for 6-well plates with100 μL TUNEL detection solution. Cover the sample with TUNEL test solution drop wise to minimize the evaporation of TUNEL test solution. After washing 3 times with PBS, the slides were mounted with anti-fluorescence quenching mounting solution, and then observed under a fluorescence microscope.

### Tumor xenografts

Five-week-old female NOD/SCID mice were used for the animal study. The xenograft mice models, including HCT 116, SW620 cells, and patient-derived xenograft (PDX), were grouped randomly. HCT 116 and SW620 cells (1 × 10^6^ cells) as well the PDX model in 100 μL of PBS were injected subcutaneously into both flanks of each mouse. When the tumors developed to a certain volume, DEH was used as follows: the control group (*N* = 3) was injected intraperitoneally with 100 μL PBS (containing 0.1% DMSO); the other groups (N = 3, per group) were injected with DEH at 40 mg/kg (mouse body weight) once every other day. The tumor volume was also measured once every 2 days. Finally, the tumors were removed from the bodies of the mice and were photographed and weighed immediately.

### IHC and H&E assays

The xenograft tumors were fixed in 3.7% paraformaldehyde (PFA) at 4 °C for 48 h. After washing with PBS, the samples were dehydrated and embedded in paraffin. The paraffin sections were deparaffinized following hydration and antigen retrieval. The sections were then incubated in 0.3% hydrogen peroxide for 15 min and subsequently incubated with Ki67 (8D5) Mouse mAb (1: 200, #9449), BiP (C50B12) Rabbit mAb (1: 100, #3177), or LC3B (D11) Rabbit mAb (1: 100, #3868) at 4 °C overnight. After washing with PBS, the sections were incubated with an anti-rabbit biotinylated antibody at room temperature for 2 h. The amplified positive signal was observed under the microscope after staining with DAB reagent (Beyotime, China). The rates of Ki67 positive signal were calculated from five randomly selected fields, and the Ki67-positive cells were subsequently quantified. Moreover, the tissues and tumors were stained with hematoxylin and eosin (H&E) (Sangon, China). All the images were obtained by microscopy.

### Statistical analysis

All the experiments were carried out in three independent technical and biological replicates. The results of the flow cytometry were analyzed by the software FlowJo according to the publisher’s instructions. Statistical analysis was performed using the program GraphPad Prism. All the acquired quantitative data were presented as mean ± standard deviation (SD). The groups were compared using the Unpaired Student’s t-test, and a *p*-value of < 0.05 was considered to be statistically significant (*p < 0.05, **p < 0.01, and ***p < 0.001).

## Results

### DEH inhibits the proliferation of colorectal cancer cells in vitro

**Fig. 1:**
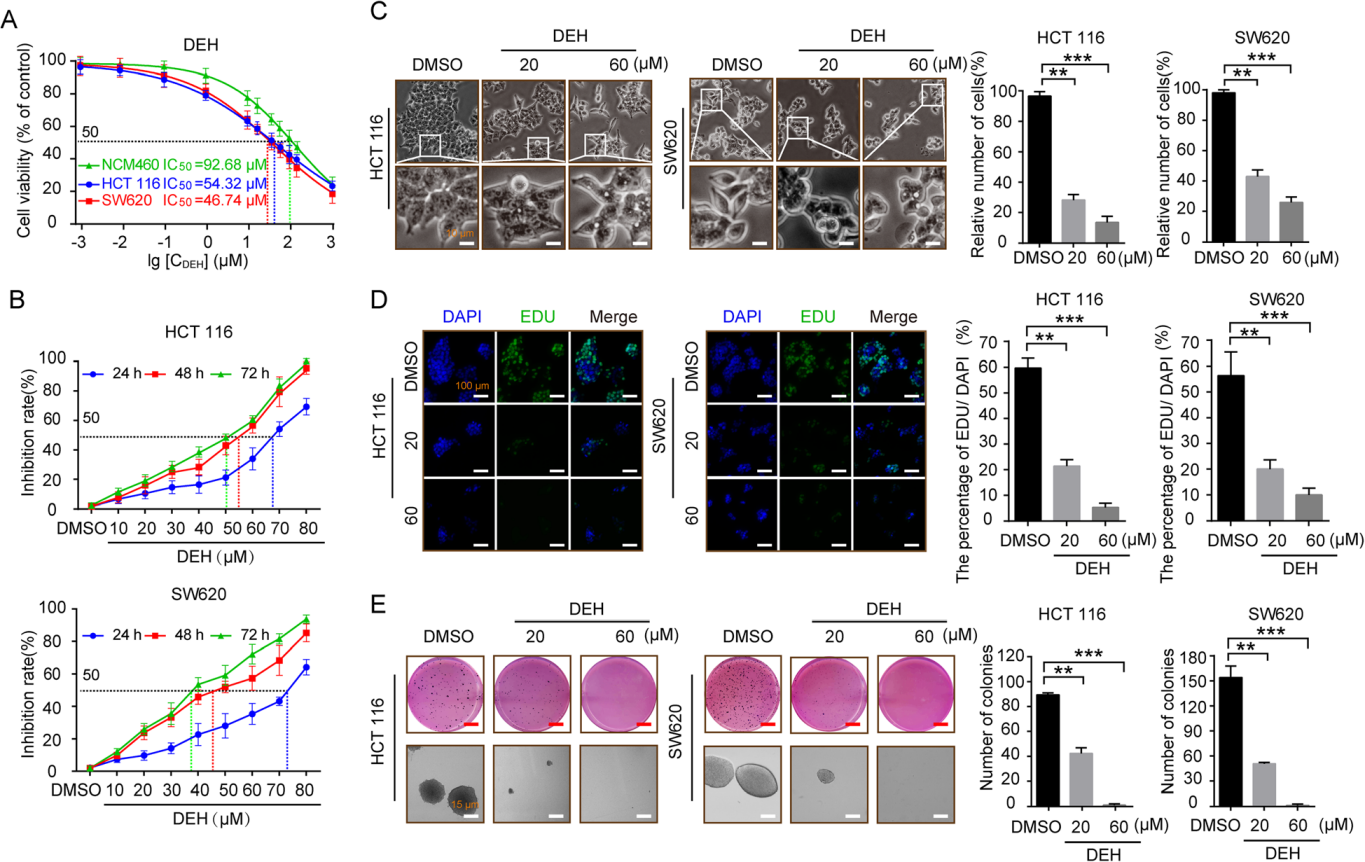
DEH inhibits the growth of colorectal cancer cells in vitro. **a** Colorectal cells (HCT 116 and SW620) and normal human colon epithelial cell NCM460 were incubated with a series of different concentrations of DEH for 48 h. Cell viability was measured by the MTT assay. The IC_50_ values of DEH for 48 h in the tested cells are marked in the lower-left corner. **b** Dose- and time-dependent effects of DEH on HCT 116 and SW620 cells. The cells were incubated DEH at different concentrations for 24, 48, and 72 h. Cell viability was measured by MTT assay. The results are represented as the means ±SD (*N* = 3). **c** Cell morphology of HCT 116 and SW620 cells after incubation with the indicated concentrations of DEH or DMSO for 48 h. Scale bar: 10 μm. The histograms represent the effect of DEH on the cell viability. **d** Images and quantification of -positive HCT 116 and SW620 cells after treatment with DEH for 48 h. Scale bar: 100 μm. **e** Colony formation and self-renewal capability were investigated by soft agar assay after incubation with DMSO, 20, or 60 μM DEH, Scale bar: 15 μm. The number of clones was counted and statistically represented as mean ± SD. The notability analysis was performed by the Unpaired Student’s t-test, and a *p*-value less than 0.05 was considered to be statistically significant. *p < 0.05, **p < 0.01, ***p < 0.001

Dehydrodiisoeugenol (DEH, CAS: 83377–50–8), known as isoeugenol or diisoeugenol, is a benzofuran-type neolignane extracted from *Myristica fragrans* Houtt, which has been prescribed in Chinese medicine for a long time [33]. The structural formula of DEH is presented in Figure S1A. To explore the effect of DEH on cancer cells and normal cells, a MTT assay was used to determine the IC_50_ of DEH on colorectal cancer cells. In this study, normal human colon epithelial cell NCM460 and two types of colorectal cancer cells, including HCT 116 and SW620, were investigated. All the detected cells were incubated with a series of different concentrations of DEH (0.001, 0.01, 0.1, 1, 10, 20, 40, 60, 80, 100, 125, and 1000 μM, DMSO treatment as the control group) for 48 h. The results indicated that even relatively low concentrations of DEH significantly inhibited the growth of cells. Furthermore, the median lethal concentration on NCM460 was significantly higher than that on HCT 116 and SW620 cells. The IC_50_ of DEH in HCT 116 and SW620 cells were 54.32 μM and 46.74 μM, respectively (Fig. 1a). To thoroughly investigate the anticancer effect of DEH on colorectal cancer cells, HCT 116 and SW620 cells were incubated with DEH of different concentrations (10, 20, 30, 40, 50, 60, 70, and 80 μM, DMSO treatment as the control group) for 24 h, 48 h, and 72 h, respectively. The results showed that DEH could inhibit colorectal cancer cell lines HCT 116 and SW620 in a time- and dose-dependent manner (Fig. 1b). After incubating with DEH, the cell numbers decreased in a dose-dependent manner, and the morphology of DEH-treated cells changed distinctly (Fig. 1c). Furthermore, EdU staining assay was utilized to evaluate the level of cell proliferation of the detected cells. The results suggested a notable decrease in the percentage of EdU-positive cells in cells incubated with DEH, compared to the control groups (Fig. 1d). The cellular activity was evaluated using the plate clone formation assay, and the results indicated that DEH could significantly inhibit the detected cellular activities in a dose-independent manner (Figure S1B). Soft agar assays were used to investigate the self-renewal capability of HCT 116 and SW620 cells that were treated with different concentrations of DEH (0, 20, 60 μM) for 48 h. The results indicated that the clones in the DEH-treated groups were smaller and fewer in number than the control groups (Fig. 1e). Collectively, these data indicated that DEH could significantly suppress the cell proliferation of colorectal cancer cells in a dose-dependent manner in vitro.

### DEH inhibits cell growth by inducing cell cycle arrest at the G1/S phase

**Fig. 2:**
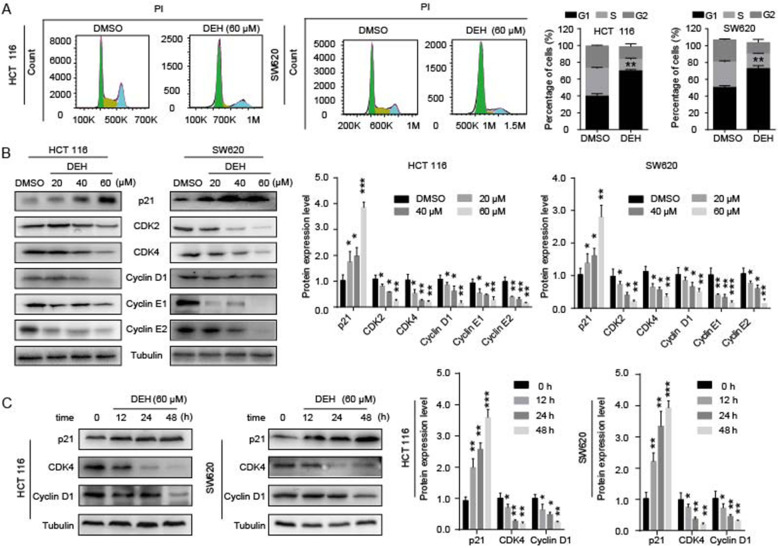
DEH inhibits cell growth by arresting the cell cycle at the G1/S phase. **a** Cell cycles of HCT 116 and SW620 cells were investigated via flow cytometry after treatment with or without DEH for 48 h. The distribution ratio of G1, S, and G2 of panel A was determined. **b** Western blotting assays were performed to detect the expression of p21, CDK2, CDK4, Cyclin D1, Cyclin E1, Cyclin E2, and Tubulin in HCT 116 and SW620 cells after treatment with DEH and the densitometry of western blotting bands of panel **c**. The protein expression levels of p21, CDK4, Cyclin D1, and Tubulin in DEH-treated colorectal cancer cells with time gradient after treatment with 60uM DEH and the densitometry of western blotting bands of panel. All the data were analyzed using the Unpaired Student’s t-test, and *p*-values less than 0.05 were considered to be statistically significant. *p < 0.05, **p < 0.01, ***p < 0.001

To understand the mechanism of cell growth and proliferation influenced by DEH, flow cytometry was used to examine the cell cycle of detected cells. There was an approximately 30% increase in the number rate of cells in the G1 phase among both the HCT 116 and SW620 cells treated with DEH, compared to control cells (Fig. 2a). The results revealed that DEH treatment caused a distinct accumulation of cells in the G1/S phase. This indicated that DEH inhibits cell growth and proliferation by inducing cell cycle arrest in the G1 phase. To further confirm the changes, we detected the expression levels of p21, CDK2, CDK4, Cyclin D1, Cyclin E1, and Cyclin E2 proteins, which could promote cells passing through the G1/S checkpoint. The results suggested that the protein expression of related cyclins and CDKs in DEH-treated cells was significantly reduced compared to counterparts in the control group cells in a dose- and time-dependent manner (Fig. 2b-c). To determine whether the decline of cell viability is caused by apoptosis to some extent, we measured the rate of apoptosis in cells by flow cytometry. However, the results indicated that there was no obvious apoptosis in cells after DEH treatment (Figure S2A). These data revealed that DEH inhibited cell growth by inducing cell cycle arrest rather than apoptosis in colorectal cancer cells.

### DEH induces autophagy in colorectal cancer cells

**Fig. 3:**
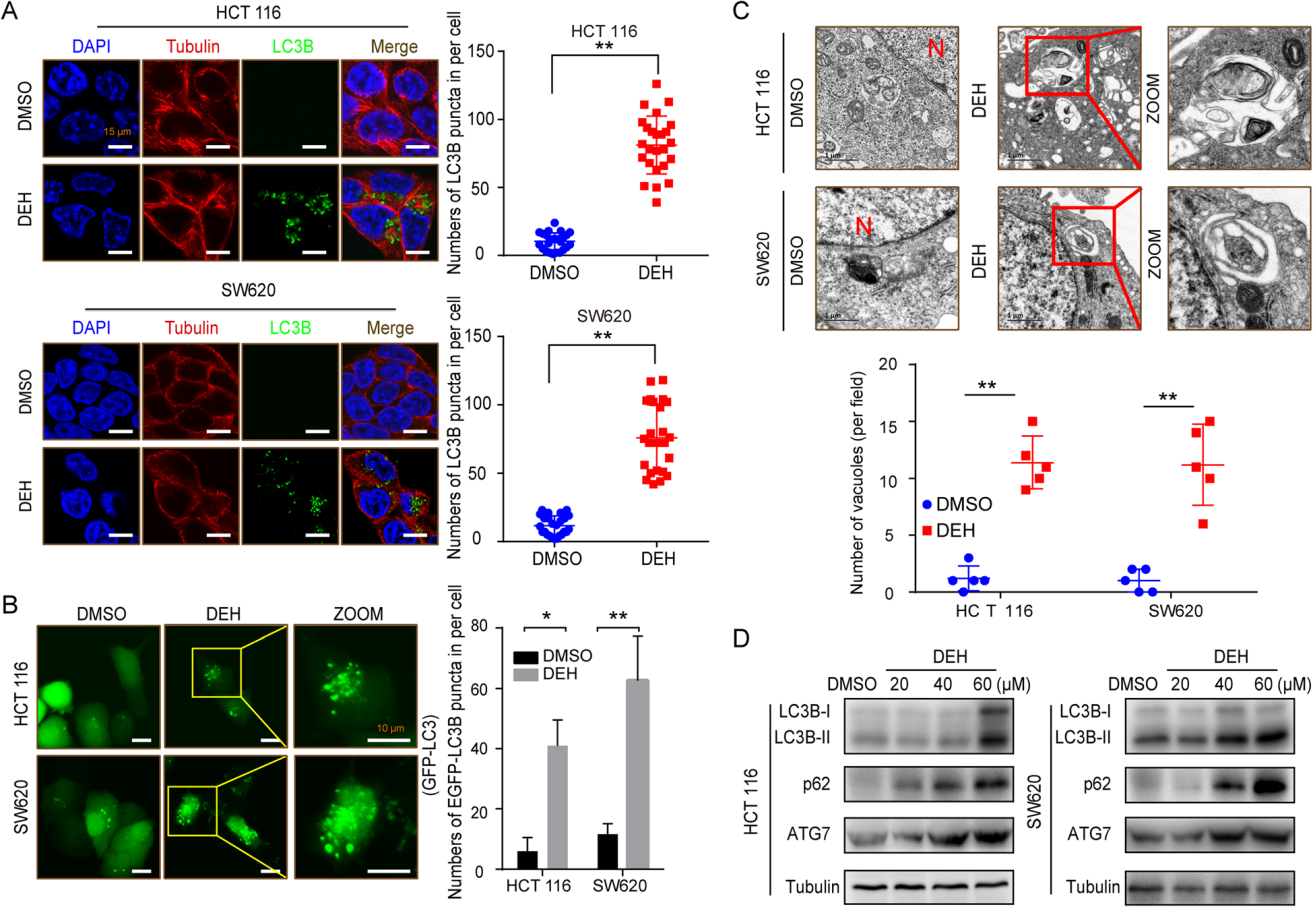
DEH induces autophagy in colorectal cancer cells. **a** Immunofluorescence staining of LC3B (green) and tubulin (red) in HCT 116 and SW620 cells treated with or without 60 μM DEH for 48 h. The nuclei were counterstained with DAPI (blue). Scale bars: 15 μm. The histogram shows quantification of the percentage of cells with LC3B puncta. **b** Fluorescence images of GFP-MAP 1LC3B puncta in HCT 116 and SW620 cells incubated with or without 60 μM DEH for 48 h. GFP-MAP 1LC3B puncta were quantified and presented in the bar chart on the right. Scale bars: 10 μm. **c** Autophagic vesicles detected by TEM in HCT 116 and SW620 cells treated with or without 60 μM DEH for 48 h. Scale bar: 1 μm. N: nucleus. **d** Protein levels of LC3B, p62, and ATG7 were detected by western blotting after HCT 116 and SW620 were treated with the indicated concentrations of DEH for 48 h. The densitometry of western blotting is shown to the right of the pane. The statistical results are presented as mean ± SD. All the data were analyzed by using the Unpaired Student’s t-test and *p*-values less than 0.05 were considered to be statistically significant. *p < 0.05, **p < 0.01, ***p < 0.001

To determine whether DEH induced cellular autophagy in colorectal cancer cells, an immunofluorescence assay was performed to check the distribution of LC3B, which is widely considered to be a marker of autophagy. The LC3B-positive cells were increased significantly in cells incubated with DEH, compared to the control group, both in HCT 116 and SW620 cells. The numbers of LC3B-positive cells were also significantly increased after DEH treatment (Fig. 3a). The formation of an autophagic vesicle was also analyzed using a GFP-tagged MAP 1LC3B expression system. The number of GFP-MAP 1LC3B-positive cells was significantly increased after incubation with DEH, compared to the control group (Fig. 3b). The autophagic vesicles eventually became autophagosomes, as observed by transmission electron microscopy (TEM). As presented in Fig. 3c, several autophagosomes were observed in HCT 116 and SW620 cells after incubation with DEH. The level of protein expression of LC3B and ATG7 were upregulated after DEH treatment in a dose- and time-dependent manner (Fig. 3d, Figure S3B). Noticeably, as a typical receptor typical of autophagy, p62 was remarkably increased after DEH treatment. This suggested that the DEH-induced autophagy in colorectal cancer was disturbed by some actions.

### DEH treatment inhibits the autophagic flux

**Fig. 4:**
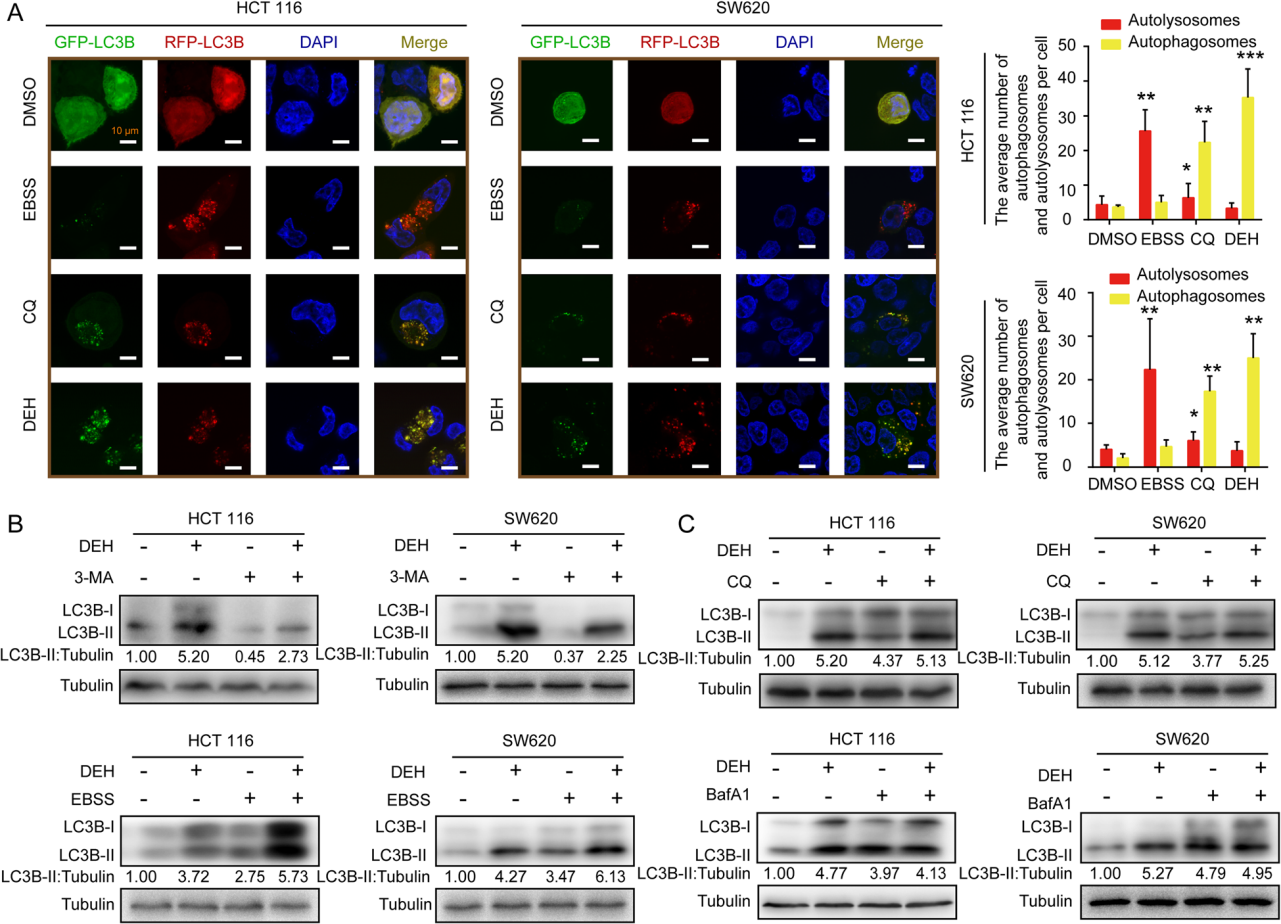
DEH inhibits autophagic flux in colorectal cancer cells. **a** Fluorescence imaging photographs of HCT 116 and SW620 cells infected with mRFP-GFP-LC3B recombinant adenovirus. The cells were infected with adenovirus for 24 h and then incubated with DMSO, EBSS, 10 μM CQ, and 60 μM DEH for 48 h. Nuclei were stained with DAPI. Scale bar: 10 μm. The average number of autophagosomes (yellow) and autolysosomes (red) per detected cell was counted. All the data were analyzed using the Unpaired Student’s t-test, and p-values less than 0.05 were considered to be statistically significant. *p < 0.05, **p < 0.01, ***p < 0.001. **b** Western blot analysis of LC3B-II levels in HCT 116 and SW620 cells incubated with or without 60 μM DEH in the absence or presence of 10 mM 3-MA for 48 h. The western blot analysis of LC3B-II levels in HCT 116 and SW620 cells incubated with or without 60 μM DEH in the absence or presence of 10 μM CQ for 48 h. **c** Western blot analysis of LC3B-II levels in HCT 116 and SW620 cells incubated in normal medium or EBSS with or without 60 μM DEH for 6 h. Western blot analysis of LC3B-II levels in HCT 116 and SW620 cells incubated with or without 60 μM DEH in the absence or presence of 10 nM Baf A1 for 48 h

The accumulation of LC3B-positive cells might result from the blockage of autophagic flux. The p62 protein was increased remarkably in cells incubated with DEH, suggesting that DEH-induced autophagy may be significantly obstructed by the inhibition of autophagic flux. To further confirm this, a GFP-RFP-LC3 double-label vector system was used in the next step. GFP and RFP were almost uniformly distributed in the DMSO-treated cells (Fig. 4a) due to the low level of background autophagy. When incubated with EBSS, the RFP puncta dominated in the starvation-treated cells. When the cells were treated with CQ, which blocks the fusion of autophagosomes with lysosomes and is widely used as a classical inhibitor of cellular autophagic flux, both the GFP and RFP puncta were almost completely coincident. The results of the control groups indicate that the GFP-RFP-LC3 double-label vector system was an effective tool to evaluate the autophagic flux. When the cells were incubated with DEH, yellow puncta dominate in the detected cells, both in the HCT 116 and SW620 cells. When the cells were incubated together with DEH and 3-methyladenine (3-MA), which is a selective inhibitor of PI3K and inhibits the formation of the early-stage autophagosome, the expression level of LC3B-II was decreased compared with the DEH-treatment alone (Fig. 4b). The expression level of LC3B-II was also analyzed after cells were incubated with DEH together, with or without CQ and Baf A1. The two compounds can inhibit the fusion of autophagosomes with lysosomes and are widely used as autophagic flux inhibitors. DEH, CQ, and Baf A1 could cause the accumulation of LC3B-II alone, while DEH incubated together with CQ or Baf A1 could not cause the further increase in the level of accumulation of LC3B-II, compared with cells treated with DEH alone (Fig. 4c). Assays were used to investigate the autophagy sinal and related protein expression level of HCT 116 and SW620 cell lines that treated with different concentrations of DEH (60 μM), (3-MA) (10 mM), CQ (10 μM) and Baf A1(10 nM) respectively. In summary, these results demonstrated that DEH could act as an inhibitor of autophagy flux to cause the accumulation of autophagosomes.

### DEH induces ER stress in colorectal cancer cells

**Fig. 5:**
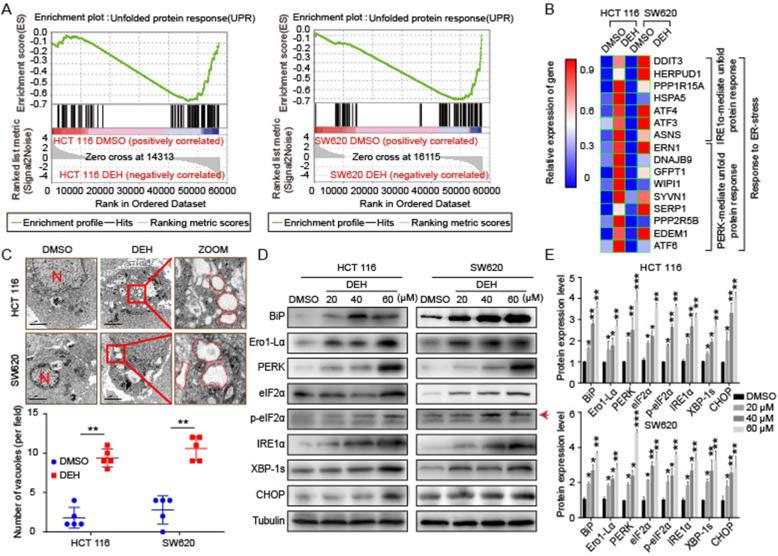
DEH induces ER stress in colorectal cancer cells. **a** Gene set enrichment analysis of UPR genes between control and DEH-treated cells. **b** The thermodynamic chart of the mRNA expression level of genes related to ER stress in colorectal carcinoma cells after incubation with DEH for 48 h. **c** The subcellular structure of colorectal cancer cells after treatment with or without 60 μM DEH for 48 h were observed by TEM. Scale bar: 2 μm. N: nucleus. The ER is circled in red. **d** Western blotting assays were performed to detect the expression of BiP, Ero1-Lα, PERK, eIF2α, p-eIF2α, IRE1α, XBP-1 s, CHOP, and Tubulin in HCT 116 and SW620 cells after treatment with or without DEH. The densitometry of western blotting in the right panel. The data were presented as means ±SD. All the data were analyzed by the Unpaired Student’s t-test and *p*-values less than 0.05 were considered to be statistically significant. *p < 0.05, **p < 0.01, ***p < 0.001

Our data demonstrated that DEH could inhibit the proliferation of colorectal cancer cells and trigger cellular autophagy via impairment of the autophagic flux. Besides, we observed that DEH treatment could induce the formation of cellular vacuoles in cells treated with DEH. The transcriptome analysis was then performed to investigate the changes of gene expression pattern in cells that were treated with DEH (60 μM) for 48 h. The unfolded protein response (UPR)-related factors were enriched thoroughly in DEH-treated cells through the GESA website (Fig. 5a). The pathways of the ER stress-related genes, PERK and IRE1α, were upregulated at the transcriptional level in both HCT 116 and SW620 cells (Fig. 5b). To confirm this phenomenon at the cellular level, transmission electron microscopy was used to intensively observe the DEH-treated colorectal cancer cells. The results indicated that more dilated cytoplasmic vacuoles were recognized as dilated ER lumens. The numerous ER stress were more distinctly identified in DEH-treated colorectal cancer cells than in the control group (Fig. 5c). To validate these results, we further examined the ER-stress-related proteins, including BiP, Ero1-Lα, PERK, eIF2α, p-eIF2α, IRE1α, XBP-1 s, and CHOP in colorectal cancer cells treated with different concentrations of DEH for 48 h. The results indicated that the expression level of the examined ERS-related proteins was increased in a dose and time-dependent manner (Fig. 5d, Figure S3C). Collectively, these data demonstrated that DEH could significantly induce ER stress in human colorectal cancer cells.

### DEH-induced autophagy is related to the ER stress-dependent activation of PERK and IRE1α

**Fig. 6:**
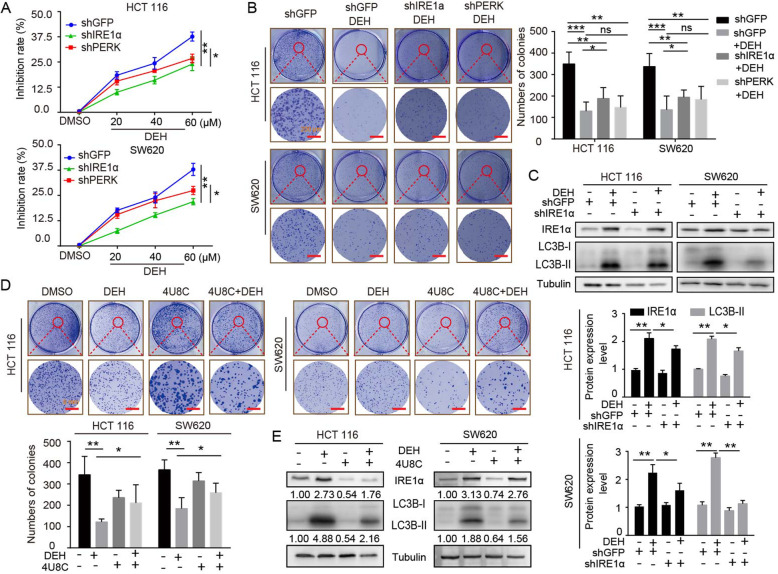
EDH induces autophagy through PERK/eIF2α and IRE1α/XBP-1 s/CHOP pathways in colorectal cancer cells. **a** The MTT assay was used to evaluate the inhibition rate of colorectal carcinoma cells, which were transfected with PERK or IRE1α siRNAs, followed by incubation with the indicated concentrations of DEH for another 48 h. **b** The cell activity was detected by colony formation assay. Cells were transfected with PERK or IRE1α siRNAs, followed by incubation with 60 μM DEH for 10 days. The cells were stained with crystal violet staining solution. Scale bar: 200 nm. The number of clones was quantitated and presented to the right of the panel. **c** The western blotting assay was used to detect the expression of IRE1α, LC3B, and Tubulin. Tubulin was used as an internal control. **d** Cellular activity was also detected by colony formation assay. The cells were pretreated with 4U8C, followed by incubation with DEH for 10 days. The cells were stained with crystal violet staining solution. Scale bar: 200 nm. The number of clones were quantified and presented below the panel. **e** The expression of IRE1α, LC3B- II, and Tubulin was detected after DEH treatment with the inhibitor of IRE1α, 4U8C, or DMSO for 48 h. All the data were analyzed using the Unpaired Student’s t-test, and p-values less than 0.05 were considered to be statistically significant. *p < 0.05, **p < 0.01, ***p < 0.001

ER stress is closely related to the activation of autophagy [34]. To understand the correlation between ER stress and autophagy induced by DEH, RNA knockdown experiments were performed. The inhibitory effect of DEH on shIRE1α-cells and shPERK-cells was considerably weaker than that of the GFP-group (Fig. 6a). A contact-dependent proliferation assay by plate cloning technique was performed to further confirm the effect of DEH on cells with interfered expression of IRE1α and PERK genes compared to the control group. Our results indicated that DEH-induced ER Stress siginificantly inhibited the proliferation and growth of colorectal cancer cells (Fig. 6b). We then checked this at the protein level using western blot analysis of the expression level of IRE1α and LC3B with tubulin as the control. The data suggested that the expression of LC3B-II protein decreased after DEH treatment along with shIRE1α, compared to the control group (Fig. 6c). Subsequently, we used the inhibitor of IRE1α to further identify the role of IRE1α in DEH-induced autophagy (Fig. 6d-e). The results were consistent with the gene interference treatment. Our findings suggest that DEH could induce ER-stress through unfolded protein responses and may inhibit autophagy by subsequently activating the PERK and IRE1α pathways.

### DEH exerts anticancer effects in vivo

**Fig. 7:**
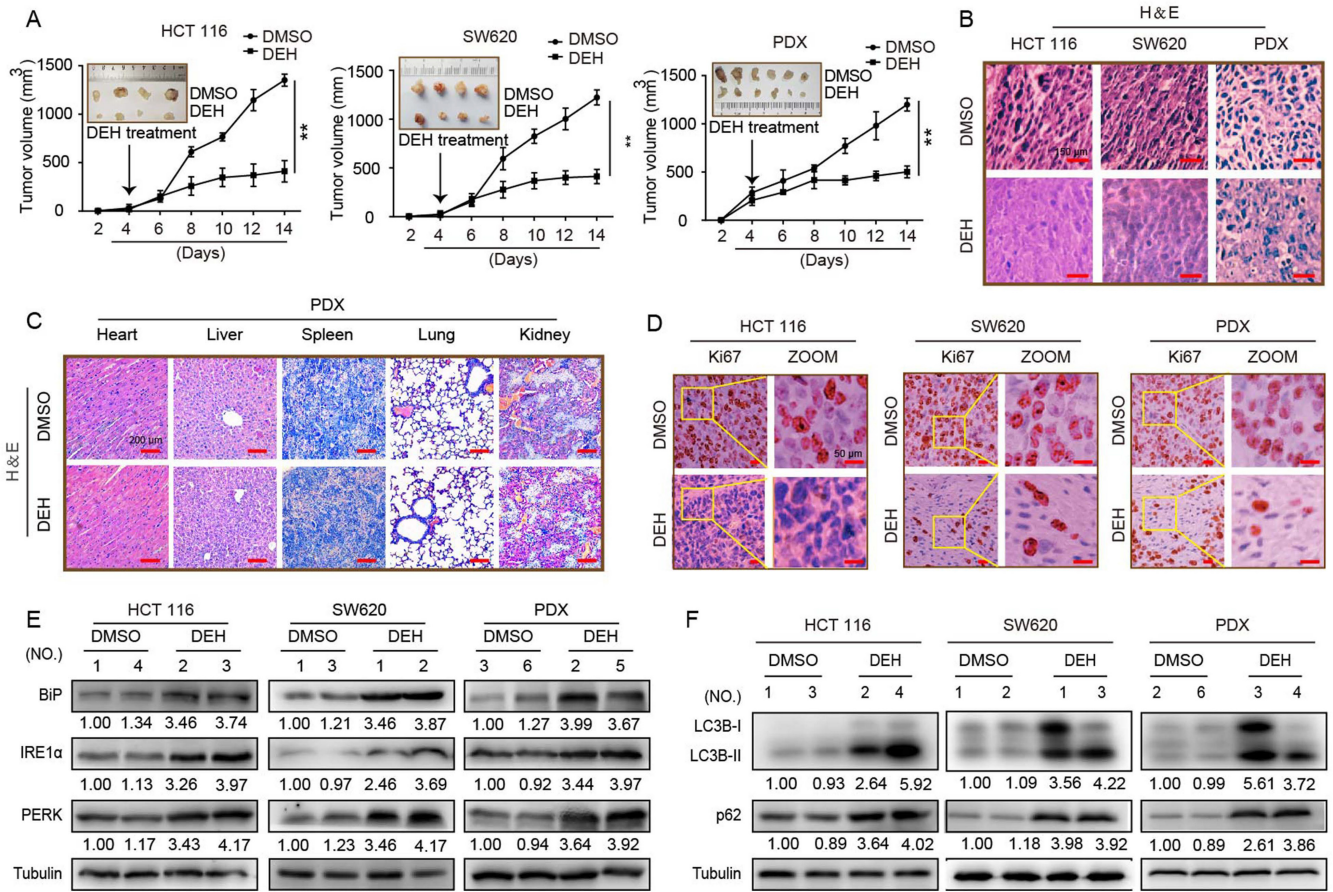
Effects of DEH on the growth of colorectal cancer in vivo. **a** HCT 116, SW620, and the tumor tissue from colon cancer patient were injected or transplanted into the flanks of NOD/SCID mice. The tumor-bearing mice were treated with DMSO or 40 mg/kg DEH by intraperitoneal injection when tumors were palpable. Tumor volume was measured every 2 days. Two weeks later, the mice were anesthetized and killed, and the tumors were imaged and analyzed. **b** Hematoxylin and eosin (H&E) staining of the indicated xenograft tumors. Scale bar: 50 μm. **c** Immunohistochemical (IHC) staining of the indicated xenograft tumors. Scale bar: 50 μm. **d** H&E staining of the heart, liver, spleen, lung, and kidney in mice treated with DMSO or 50 mg/kg DEH. Scale bar: 50 μm. **e** The expression of BiP, PERK, and IRE1α of xenograft tumors was detected by western blotting. **f** The expression of LC3B, p62, and tubulin in xenograft tumors was detected by western blotting. All the data were presented as means ±S.D. and are representative of three independent experiments. *P*-value < 0.05 was considered to be significant. **P < 0.01; ***P < 0.001

To evaluate the effects of DEH on the tumorigenic ability of colorectal cancer cells, we performed a subcutaneous tumor experiment using cell-derived xenograft (CDX) and patient-derived tumor xenograft (PDX) models in vivo. The tumor size, weight, and growth rate were all significantly decreased in the DEH-treated group compared to the control group (Fig. 7a, Figure S4A). Furthermore, the percentage of Ki67-positive cells was significantly rEdUced in the DEH-treated group compared to the control group (Fig. 7c, Figure S4B). that DEH also inhibited tumor growth in vivo. Importantly, DEH had no remarkable effect on the body weight of all the tested mice (Figure S4C). Next, we examined the heart, liver, spleen, lung, and kidney of mice treated with DEH or DMSO using H&E staining. DEH treatment did not appear to have a significant effect on the pathological features of the organs of measuring mice (Fig. 7d), indicating that the drug is safe in mice. Moreover, H&E staining was used to detect the tumors in the DEH-treated and control groups (Fig. 7b). The results indicated that DEH barely had any toxic and adverse effects in experimental mice. Meanwhile, the magnitude of tumors was largely decreased in the DEH treatment group. All the data confirm that DEH has significant inhibitory effects on the tumorigenesis and growth of colorectal cancer, with low-toxicity.

### DEH induces ER stress and inhibits autophagic flux in vivo

We homogenized the tumor from injected mice and again detected the related proteins by western blot assays. The results indicated that the expression level of ER stress-related proteins, including BiP, PERK, and IRE1α was notably upregulated in colorectal cancer after DEH treatment (Fig. 7e). The expression level of autophagy-related proteins in tumor tissues also increased significantly after treatment with DEH, compared to the control group (Fig. 7f). These results at an individual level were largely consistent with the cellular-level. Collectively, these findings implied that DEH-induced autophagy could be significantly disturbed by the DEH-induced ER-stress, which also indicates that DEH displays an excellent anti-tumor effect in vivo.

## Discussion

Despite the emergence of numerous therapeutic and screening applications, colorectal cancer remains a major life-threatening malignancy [35, 36]. Unfortunately, patients with advanced or recurrent colorectal cancer usually have a low survival rate due to drug toxicity of chemotherap or side-effects of radiotherapy after common treatments for colorectal cancer [37]. Therefore, it is critical to actively explore the high-efficiency and low-toxicity drugs for patients with poor prognosis after surgery to improve their quality of life [38, 39]. Presently, the treatment of diseases with traditional Chinese medicine has shown great potential [40]. Chinese traditional medicine has been widely known to have a long history in the treatment of several diseases, and the research on monomers isolated from traditional Chinese medicines has also become increasingly prevalent [41, 42]. Moreover, traditional Chinese medicine has tremendous potential for the treatment of cancer owing to its extremely low toxicity over its long course of application [43]. Nutmeg, the seed of the evergreen tree plant *Myristica fragrans Houtt*, is an example of such traditional Chinese medicine, which has proven its potential ability in the treatment of gastric cancer, lung cancer, skin tumors, melanoma, osteosarcoma, and leukemia [44, 45]. DEH, a type of highest-acquired active monomer, is extracted from nutmeg. Recent studies have demonstrated that DEH exerts fantastic anti-inflammatory and anti-allergic effects in several digestive disorders [46, 47]. However, research on the effects of DEH in colorectal cancer has not been conducted till now.

Here, we described the critical role of DEH in the treatment of colorectal cancer and explored the relevant mechanisms for its possible therapeutic application. In this study, we evaluated the anti-tumor activity of DEH in colorectal cancer. First, we demonstrated through the MTT and EdU assays that DEH significantly inhibited cell proliferation and growth in a dose- and time-dependent manner in vitro. The soft agar assay revealed that the colony size was smaller after DEH treatment than control groups. Moreover, the flow cytometry analysis indicated that DEH inhibited cell growth in a time- and dose-dependent manner by arresting the cell cycle at the G1/S phase. Furthermore, the results suggested that DEH could not induce apoptosis in colorectal cancer cells.

Based on our understanding of the physiological function of autophagy, we now know that a basic level of autophagy and moderate levels of stress-induced autophagy may be important to maintain cellular metabolism [46, 47]. However, there was no definite evidence of the specific role and physiological functions of autophagy in different cell microenvironments [48]. Besides, uncontrolled autophagy or exceptionally blocked autophagy flux will reduce the ability to clear intracellular proteins and damaged organelles, resulting in genomic instability; thus, inhibiting cell proliferation and growth [46, 47]. Our results demonstrated that DEH could significantly induce autophagy signal in colorectal cancer cells, which was also confirmed by TEM observations. To further explore the mechanism of this phenomenon, we also analyzed the expression of autophagy-related proteins again. p62 protein was upregulated by DEH treatment, suggesting that the DEH-induced blockage of autophagy had a devastating effect on cells, the mechanism of which needs to be investigated in detail. Then, the GFP-RFP-LC3 double-label vector system was used to detect the flow of autophagy induced by DEH. The results confirmed that the autophagic flow was blocked. The autophagy inhibition induced by DEH may be due to the impaired degradation process of autophagosomes. Next, we combined the autophagy activator and with its corresponding inhibitor to detect the expression level of proteins in DEH-treatment cells by western blot. The results confirmed that the inhibition of autophagy may occur in the later stages of autophagy. The blockage of the autophagic flow inhibits the growth and proliferation of cells. This has made it possible to treat cancer using the autophagy-inhibition strategy in cancer cells. This may be due to the enhanced autophagy of proteins. Finally, DEH could further inhibit the growth of colorectal cancer cells through the inhibition of autophagy.

In our study, microscopic observation and transcriptome data analysis indicated that DEH also caused strong ER stress. Then, the TEM observations combined with western blotting assay were used to confirm that DEH caused ER stress in colorectal cancer cells in a dose- and time-dependent manner. According to previous reports, ER stress can not only increase autophagy but also inhibit it [46, 47]. Besides, stress-mediated autophagy has been UPR [49] and its downstream pathways.

To elucidate this signaling pathway, we silenced two key genes of the ER stress pathway and then treated the cells again with drugs [50]. Through MTT and plate-cloning experiments, we observed that the DEH-induced inhibition of autophagy was activated by DEH-induced ER stress. However, the of DEH-induced stress activates the inhibition of autophagy [51] remains to be further explored.

**Fig. 8:**
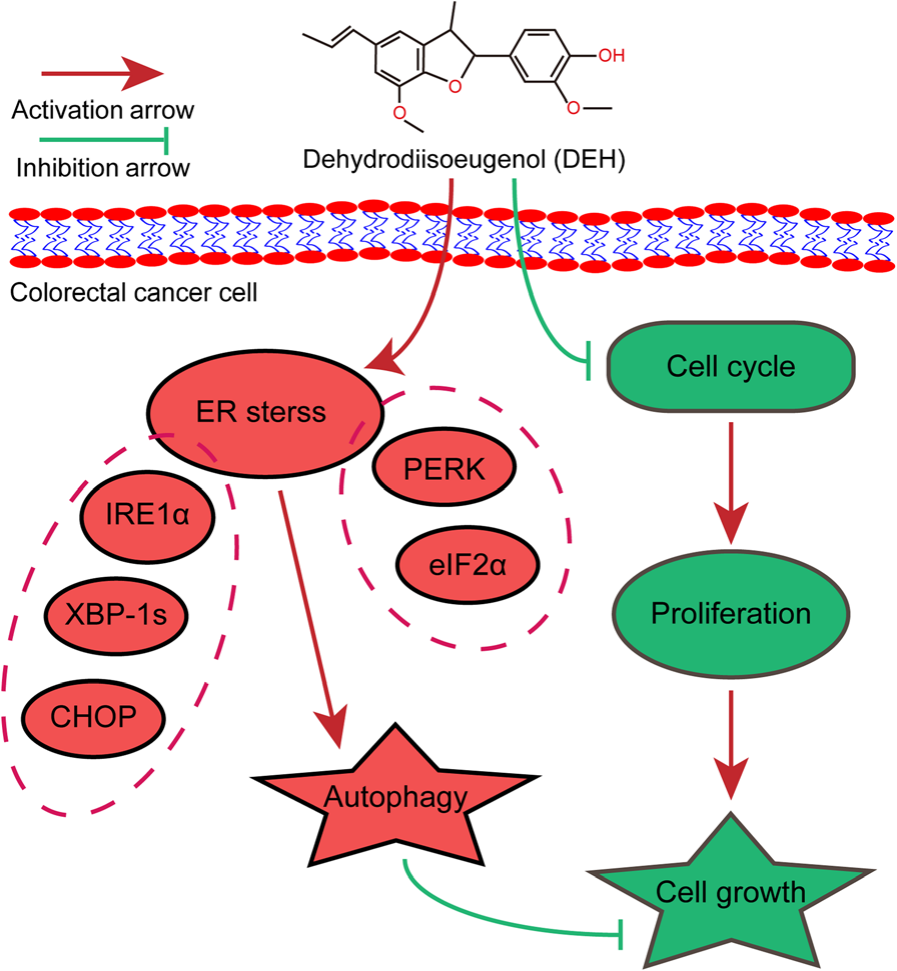
Diagram of the predicted mechanism of DEH treatment in colorectal cancer. Dehydrodiisoeugenol (DEH), as the monomer extracted from the Nutmeg seeds, showing an excellent anti-tumor activity to colorectal cancer both in vivo and in vitro. On the one hand, DEH can inhibit the proliferation of colorectal cancer cells via arresting cell cycle in a dose- and time-dependent manner. On the other hand, DEH cause endoplasmic reticulum (ER) stress and subsequently stimulates autophagy inhibition through the PERK-eIF2α / IRE1α-XBP-1 s-CHOP signal pathways. So as to inhibit the cell growth proliferation of colorectal cancer more effectively. Taken together, our studies suggest that DEH may be a promising therapeutic agent against colorectal cancer in the future

Subsequently, the xenograft experiments with CDX and PDX models were used to check the anti-cancer effect of DEH at the individual level. The growth of tumors formed in nude mice subcutaneously grew slowly after DEH treatment, with the weight and volume of the tumors formed in nude mice being significantly slower and smaller after DEH treatment, despite there being negligible variation in the weight and volume of the mice. These results suggest that DEH could inhibit the growth of colorectal cancer in vivo and in vitro*,* even at an individual level. Furthermore, the toxicity of DEH was low, and it was found to be safe in mice. We also evaluated the expression level of autophagy-related proteins and ER stress-related proteins in tumors from the tested mice. The results were consistent with those at the cellular level. Accordingly, our study on DEH may provide a promising therapeutic agent for the treatment of colorectal cancer. Overall, our study has demonstrated that DEH could inhibit cell growth and proliferation, as well as induce ERS-autophagy to exert the obvious anti-cancer effects on colorectal cancer cells (Fig. 8). This role of DEH in colorectal cancer may represent a novel treatment strategy for patients. Therefore, it is clear that DEH exerts a wonderful anticancer activity on colorectal cancer, with low toxicity.

## Conclusions

In conclusion, our data demonstrate the novel anticancer mechanism of DEH in human colorectal cancer by arresting the cell cycle in a dose- and time-dependent manner and blocking autophagy via ER stress, which could effectively inhibit the growth of colorectal cancer. These findings suggest that DEH may be a promising therapeutic drug for the treatment of colorectal cancer.

## Supplementary Information

**Additional file 1: Figure S1.** DEH inhibits cell growth of colorectal cancer cells, but not through apoptosis. A. The chemical structure of DEH used in this study. B. The plate colony formation assay was used to evaluate the cellular activity after treatment with DMSO or DEH. The numbers of clone were quantified and shown on the right of the panel. C. Cell cycles of HCT 116 and SW620 cells were investigated via flow cytometry after treatment with or without DEH for 24 h and 72 h. The distribution ratio of G1, S, and G2 of panel C was determined. All the data are means ±S.D. and are representative of three independent experiments. *P*-value less than 0.05 was considered to be statistically significant. ns: there was no significant difference, ***P < 0.001.

**Additional file 2: Figure S2.** DEH could not induces apoptosis in colorectal cancer cells. A. HCT 116 and SW620 cells were incubated with DMSO or DEH for days, and the cell apoptosis was demonstrated by Annexin V/PI staining with flow cytometry. The cell apoptosis statistics were listed to the right of the panel. B. The immunostaining of TUNEL cell apoptosis detection was also used with DEH (60 μM), and the 5-FU (2 μM) was used as an positive control. The quantification was shown on the below of the panel. All the data are means ±S.D. and are representative of three independent experiments. P-value less than 0.05 was considered to be statistically significant. ns: there was no significant difference, ***P < 0.001.

**Additional file 3: Figure S3.** Expression profiles of autophagic and associated genes of colorectal cancer cells after treatment with DMSO or DEH. A. A heatmap of autophagic and associated genes of HCT 116 and SW620 cells after incubation with DMSO or DEH for 2 days. B. Western blotting was performed to investigate the expression of LC3B, p62, ATG7, and Tubulin in HCT 116 and SW620 cells after treatment with DEH. C. Western blotting was performed to investigate the expression of BiP, PERK, IRE1α, and Tubulin in HCT 116 and SW620 cells after treatment with DEH. Tubulin was used as a control. D. Images and quantification of -positive HCT 116 and SW620 cells after DEH treatment with PERK or IRE1α siRNAs for 48 h. Scale bar: 100 μm. Scale bar: 15 μm. The number of clones was counted and statistically represented as mean ± SD. The notability analysis was performed by the Unpaired Student’s t-test, and a *p*-value less than 0.05 was considered to be statistically significant. *p < 0.05, **p < 0.01, ***p < 0.001.

**Additional file 4: Figure S4.** The anticancer activities of DEH on colorectal cancer were evaluated using CDX and PDX models in vivo. A. Quantified results of tumor weight. B. The Ki67-positive cells of tumor-bearing mice after treatment with DMSO or DEH were quantified and shown in the bar chart. C. The weights of the tumor-bearing mice were measured after treatment with DMSO or DEH for 2 weeks. All the data are presented as means ±S.D. and represent three independent experiments. *P*-value < 0.05 was considered to be significant. **P < 0.01; ***P < 0.001.

**Additional file 5: Figure S5.** The ethics review.

## Data Availability

All the data reported by the manuscript are publicly available and the materials are also freely available.

## References

[1] Gao C, Cao W, Bao L, Zuo W, Xie G, Cai T, Fu W, Zhang J, Wu W, Zhang X (2010). Autophagy negatively regulates Wnt signalling by promoting Dishevelled degradation. Nat Cell Biol.

[2] Levy JMM, Towers CG, Thorburn A (2017). Targeting autophagy in cancer. Nat Rev Cancer.

[3] Rogov V, Dötsch V, Johansen T, Kirkin V (2014). Interactions between autophagy receptors and ubiquitin-like proteins form the molecular basis for selective autophagy. Mol Cell.

[4] Kim I, Xu W, Reed JC (2008). Cell death and endoplasmic reticulum stress: disease relevance and therapeutic opportunities. Nat Rev Drug Discov.

[5] Shimodaira Y, Takahashi S, Kinouchi Y, Endo K, Shiga H, Kakuta Y, Kuroha M, Shimosegawa T (2014). Modulation of endoplasmic reticulum (ER) stress-induced autophagy by C/EBP homologous protein (CHOP) and inositol-requiring enzyme 1α (IRE1α) in human colon cancer cells. Biochem Biophys Res Commun.

[6] Li T, Su L, Zhong N, Hao X, Zhong D, Singhal S, Liu X (2013). Salinomycin induces cell death with autophagy through activation of endoplasmic reticulum stress in human cancer cells. Autophagy.

[7] Høyer-Hansen M, Jäättelä M (2007). Differentiation: Connecting endoplasmic reticulum stress to autophagy by unfolded protein response and calcium. Cell Death Differ.

[8] Krishnamachary B, Berg-Dixon S, Kelly B, Agani F, Feldser D, Ferreira G, Iyer N, LaRusch J, Pak B, Taghavi P (2003). Regulation of colon carcinoma cell invasion by hypoxia-inducible factor 1. Cancer Res.

[9] Smeby J, Sveen A, Merok MA, Danielsen SA, Eilertsen IA, Guren M, Dienstmann R, Nesbakken A, Lothe RA (2018). CMS-dependent prognostic impact of KRAS and BRAF V600E mutations in primary colorectal cancer. Ann Oncol.

[10] X-z C, Z-y C, Liao L-m, Liu Z-Z, Du J (2014). Application of serum pharmacology in evaluating the antitumor effect of Fuzheng Yiliu Decoction from Chinese medicine. Chin J Integr Med.

[11] Wang S-Y, Shiao M-SJJoF, Analysis D. Pharmacological functions of Chinese medicinal fungus Cordyceps sinensis and related species. J Food Drug Anal. 2000;4(2000):248–57.

[12] Li F, Yang XW (2012). Analysis of anti-inflammatory dehydrodiisoeugenol and metabolites excreted in rat feces and urine using HPLC-UV. Biomed Chromatogr.

[13] Murakami Y, Shoji M, Hirata A, Tanaka S, Yokoe I, Fujisawa S (2005). Dehydrodiisoeugenol, an isoeugenol dimer, inhibits lipopolysaccharide-stimulated nuclear factor kappa B activation and cyclooxygenase-2 expression in macrophages. Arch Biochem Biophys.

[14] Yang A-H, He X, Chen J-X, He L-N, Jin C-H, Wang L-L, Zhang F-L, An LJ (2015). Identification and characterization of reactive metabolites in myristicin-mediated mechanism-based inhibition of CYP1A2. Chem Biol Interact.

[15] Fang Z, Gong C, Yu S, Zhou W, Hassan W, Li H, Wang X, Hu Y, Gu K, Chen X (2018). NFYB-induced high expression of E2F1 contributes to oxaliplatin resistance in colorectal cancer via the enhancement of CHK1 signaling. Cancer Lett.

[16] Choi JH, Won YW, Kim HS, Oh YH, Lim S, Kim HJ (2016). Oxaliplatin-induced sinusoidal obstruction syndrome mimicking metastatic colon cancer in the liver. Oncol Lett.

[17] Al-Rawi SS, Ibrahim AH, Ab Rahman NNN, Nama MMB, Majid AMA, Ab Kadir MO. The effect of supercritical fluid extraction parameters on the nutmeg oil extraction and its cytotoxic and antiangiogenic properties. Procedia Food Science. 2011;1:1946–52.

[18] Zhou L, Zhang Z, Huang Z, Nice E, Zou B, Huang C. Revisiting cancer hallmarks: insights from the interplay between oxidative stress and non-coding RNAs.Mol Biomed. 2020;1(1):1–24. 10.1186/s43556-020-00004-1 PMC8603983 35006436

[19] Simmonds P, Best L, George S, Baughan C, Buchanan R, Davis C, Fentiman I, Gosney M, Northover J, Williams C. Surgery for colorectal cancer in elderly patients: a systematic review. Lancet. 2000;356(9234):968–74. 11041397

[20] Li F, Yang X-W, Krausz KW, Nichols RG, Xu W, Patterson AD, Gonzalez FJ (2015). Modulation of colon cancer by nutmeg. J Proteome Res.

[21] ​Schepetkin IA, Kushnarenko SV, Özek G, Kirpotina LN, Sinharoy P, Utegenova GA, Abidkulova KT, Özek T, Başer KHC, Kovrizhina AR, Khlebnikov AI, Damron DS, Quinn MT. Modulation of human neutrophil responses by the essential oils from Ferula akitschkensis and their constituents. J Agr Food Chem. 2016;64(38):7156–70. 10.1021/acs.jafc.6b03205 PMC5048753 27586050

[22] Sepulveda AR, Hamilton SR, Allegra CJ, Grody W, Cushman-Vokoun AM, Funkhouser WK, Kopetz SE, Lieu C, Lindor NM, Minsky BD (2017). Molecular biomarkers for the evaluation of colorectal cancer: guideline from the American society for clinical pathology, College of American Pathologists, Association for Molecular Pathology, and American Society of Clinical Oncology. J Clin Oncol.

[23] El-Alfy AT, Wilson L, MA ES, Abourashed EA. Towards a better understanding of the psychopharmacology of nutmeg: activities in the mouse tetrad assay. J Ethnopharmacol. 2009;126(2):280–6. 10.1016/j.jep.2009.08.026 PMC2783227 19703539

[24] Mikhaylova O, Stratton Y, Hall D, Kellner E, Ehmer B, Drew AF, Gallo CA, Plas DR, Biesiada J, Meller J. VHL-regulated MiR-204 suppresses tumor growth through inhibition of LC3B-mediated autophagy in renal clear cell carcinoma. Cancer Cell. 2012;21(4):532–46. 10.1016/j.ccr.2012.02.019 PMC3331999 22516261

[25] Prakash E, Gupta DK. Cytotoxic activity of ethanolic extract of *Myristica fragrans* (Houtt) against seven human cancer cell lines. J Food Nutr Sc. 2013;1(1):1–3.

[26] Wang F, Jiang J, Hu S, Ma H, Zhu H, Tong Q, Cheng L, Hao X, Zhang G, Zhang Y (2017). Secondary metabolites from endophytic fungus Chaetomium sp induce colon cancer cell apoptotic death. Fitoterapia.

[27] Yin C, Ke X, Zhang R, Hou J, Dong Z, Wang F, Zhang K, Zhong X, Yang L, Cui H (2019). G9a promotes cell proliferation and suppresses autophagy in gastric cancer by directly activating mTOR. FASEB J.

[28] Li C, Deng C, Pan G, Wang X, Zhang K, Dong Z, Zhao G, Tan M, Hu X, Shi S (2020). Lycorine hydrochloride inhibits cell proliferation and induces apoptosis through promoting FBXW7-MCL1 axis in gastric cancer. J Exp Clin Cancer Res.

[29] Yang J, Dong Z, Ren A, Fu G, Zhang K, Li C, Wang X, Cui HJ. Medicine M. Antibiotic tigecycline inhibits cell proliferation, migration and invasion via down-regulating CCNE2 in pancreatic ductal adenocarcinoma. J Cell Mol Med. 2020;24(7):4245–60. 10.1111/jcmm.15086 PMC7171345 32141702

[30] Winawer SJ, Fletcher RH, Miller L, Godlee F, Stolar M, Mulrow C, Woolf S, Glick S, Ganiats T, Bond JH (1997). Colorectal cancer screening: clinical guidelines and rationale. Gastroenterology.

[31] Moscat J, Diaz-Meco MT. p62 at the crossroads of autophagy, apoptosis, and cancer. Cell. 2009;137(6):1001–4. 10.1016/j.cell.2009.05.023 PMC3971861 19524504

[32] Li C, Zhang K, Pan G, Zhang L, Hu X, Zhao G, Deng C, Tan M, Li C, Xu M (2020). Bmintegrin β1: A broadly expressed molecule modulates the innate immune response of *Bombyx mori*. Dev Comp Immunol.

[33] Lv Q-Q, Yang X-N, Yan D-M, Liang W-Q, Liu H-N, Yang X-W, Li F, Analysis B (2017). Metabolic profiling of dehydrodiisoeugenol using xenobiotic metabolomics. J Pharm Biomed Anal.

[34] Codogno P, Meijer A. Autophagy: a potential link between obesity and insulin resistance. Cell Metab. 2010;11(6):449–51. 10.1016/j.cmet.2010.05.006 20519116

[35] Rabinowitz JD, White E (2010). Autophagy and metabolism. Science.

[36] Levine B, Kroemer G (2008). Autophagy in the pathogenesis of disease. Cell.

[37] Maheswari U, Ghosh K, Sadras SR (2018). Licarin A induces cell death by activation of autophagy and apoptosis in non-small cell lung cancer cells. Apoptosis.

[38] Pettersen K, Andersen S, van der Veen A, Nonstad U, Hatakeyama S, Lambert C, Lach-Trifilieff E, Moestue S, Kim J, Grønberg B (2020). Autocrine activin A signalling in ovarian cancer cells regulates secretion of interleukin 6, autophagy, and cachexia. J Cachexia Sarcopenia Muscle.

[39] New M, Tooze S. The Role of Autophagy in Pancreatic Cancer—Recent Advances. Biology. 2020;9(1):7. 10.3390/biology9010007 PMC7169408 31905604

[40] Zhang Z, Zhou L, Xie N, Nice EC, Zhang T, Cui Y, Huang C (2020). Overcoming cancer therapeutic bottleneck by drug repurposing. Signal Transduct Target Ther.

[41] von Haehling S, Morley JE, Coats AJ, Anker SD (2017). Ethical guidelines for publishing in the Journal of Cachexia, Sarcopenia and Muscle: update 2017. J Cachexia Sarcopenia Muscle.

[42] Pal M, Febbraio MA, Whitham M (2014). From cytokine to myokine: the emerging role of interleukin-6 in metabolic regulation. Immunol Cell Biol.

[43] Lach-Trifilieff E, Minetti GC, Sheppard K, Ibebunjo C, Feige JN, Hartmann S, Brachat S, Rivet H, Koelbing C, Morvan F (2014). An antibody blocking activin type II receptors induces strong skeletal muscle hypertrophy and protects from atrophy. Science.

[44] Shintani T, Klionsky DJ. Autophagy in health and disease: a double-edged sword. Science. 2004;306(5698):990–5. 10.1126/science.1099993 PMC1705980 15528435

[45] Zhang K, Fu G, Pan G, Li C, Shen L, Hu R, Zhu S, Chen Y, Cui H (2018). Demethylzeylasteral inhibits glioma growth by regulating the miR-30e-5p/MYBL2 axis. Cell Death Dis.

[46] Daniels MS, Bannon SA, Mork ME (2017). Frequency of germline BRCA1/2 mutations in unselected patients with colorectal cancer. J Clin Oncol.

[47] Towers CG, Wodetzki D, Thorburn A. Autophagy and cancer: Modulation of cell death pathways and cancer cell adaptations Autophagy and cancer. J Cell Biol. 2020;219(1). 10.1083/jcb.201909033 PMC7039213 31753861

[48] Xu DQ, Wang Z, Wang CY, Zhang DY, Wan HD, Zhao ZL, Gu J, Zhang YX, Li ZG, Man KY (2016). PAQR 3 controls autophagy by integrating AMPK signaling to enhance ATG 14L-associated PI 3K activity. EMBO J.

[49] Xu D, Wang Z, Chen Y (2016). Two-layer regulation of PAQR3 on ATG14-linked class III PtdIns3K activation upon glucose starvation. Autophagy.

[50] Xu D, Li X, Shao F, Lv G, Lv H, Lee J-H, Qian X, Wang Z, Xia Y, Du L (2019). The protein kinase activity of fructokinase A specifies the antioxidant responses of tumor cells by phosphorylating p62. Sci Adv.

[51] Liu J, Wang T, Creighton CJ, Wu S-P, Ray M, Janardhan KS, Willson CJ, Cho S-N, Castro PD, Ittmann MM (2019). JNK 1/2 represses Lkb 1-deficiency-induced lung squamous cell carcinoma progression. Nat Commun.

