# Correction: Dehydrodiisoeugenol inhibits colorectal cancer growth by endoplasmic reticulum stress-induced autophagic pathways

**DOI:** 10.1186/s13046-023-02733-x

**Published:** 2023-06-24

**Authors:** Changhong Li, Kui Zhang, Guangzhao Pan, Haoyan Ji, Chongyang Li, Xiaowen Wang, Xin Hu, Ruochen Liu, Longfei Deng, Yi Wang, Liqun Yang, Hongjuan Cui

**Affiliations:** 1 State Key Laboratory of Silkworm Genome Biology, College of Sericulture, Textile and Biomass Sciences, Southwest University, #2, Tiansheng Rd., Beibei District, , Chongqing, 400716 China; 2 Cancer Centre, Medical Research Institute, Southwest University, Chongqing, 400716 China; 3 Affiliated Hospital of Southwest University (the Ninth People’s Hospital of Chongqing), Chongqing, 400716 China

**Correction: J Exp Clin Cancer Res 40, 125 (2021)**

**https://doi.org/10.1186/s13046-021-01915-9**

Following publication of the original article [1], the authors identified minor errors in Figs. 2, 5, 6, and 7, specifically:

• Fig. 2: p21/SW620 in 2B&C, Tub/HCT116 and CDK4/SW620 in 2B
• Fig. 5: CHOP/HCT116, BiP/SW620 in 5D
• Fig. 6: shIRE1α/DEH& shPERK/DEH of HCT116 in 6B
• Fig. 7: DEH/HCT116 in 7D, p62&Tub/SW620 and p62/PDX in 7F

The corrected figures are given below. This correction does not have any effect on the result, or conclusions of the paper. The original article has been corrected.

**Fig. 2:**
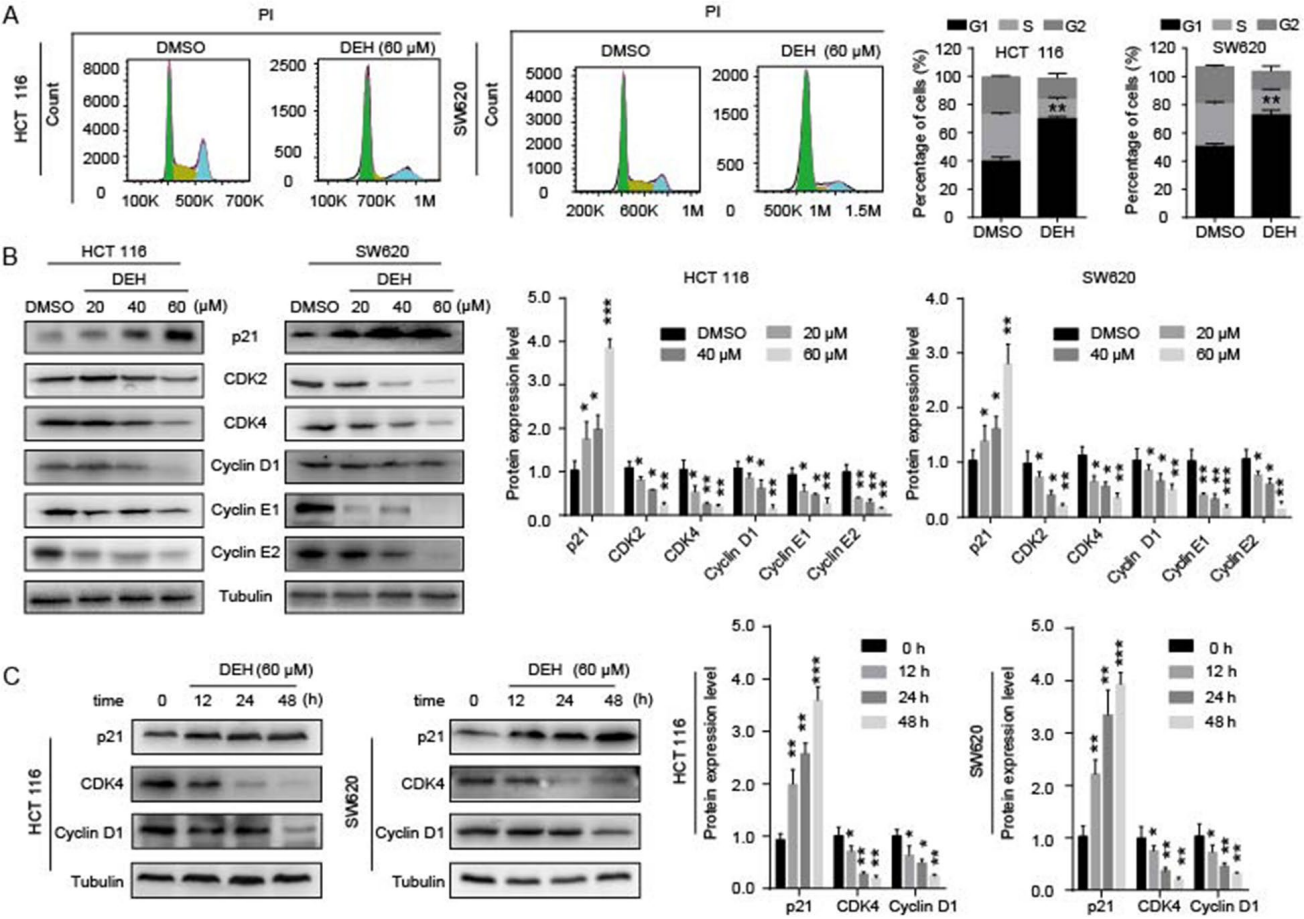
DEH inhibits cell growth by arresting the cell cycle at the G1/S phase. **a** Cell cycles of HCT 116 and SW620 cells were investigated via flow cytometry after treatment with or without DEH for 48 h. The distribution ratio of G1, S, and G2 of panel A was determined. **b** Western blotting assays were performed to detect the expression of p21, CDK2, CDK4, Cyclin D1, Cyclin E1, Cyclin E2, and Tubulin in HCT 116 and SW620 cells after treatment with DEH and the densitometry of western blotting bands of panel **c**. The protein expression levels of p21, CDK4, Cyclin D1, and Tubulin in DEH-treated colorectal cancer cells with time gradient after treatment with 60uM DEH and the densitometry of western blotting bands of panel. All the data were analyzed using the Unpaired Student’s t-test, and *p*-values less than 0.05 were considered to be statistically significant. *p < 0.05, **p < 0.01, ***p < 0.001

**Fig. 5:**
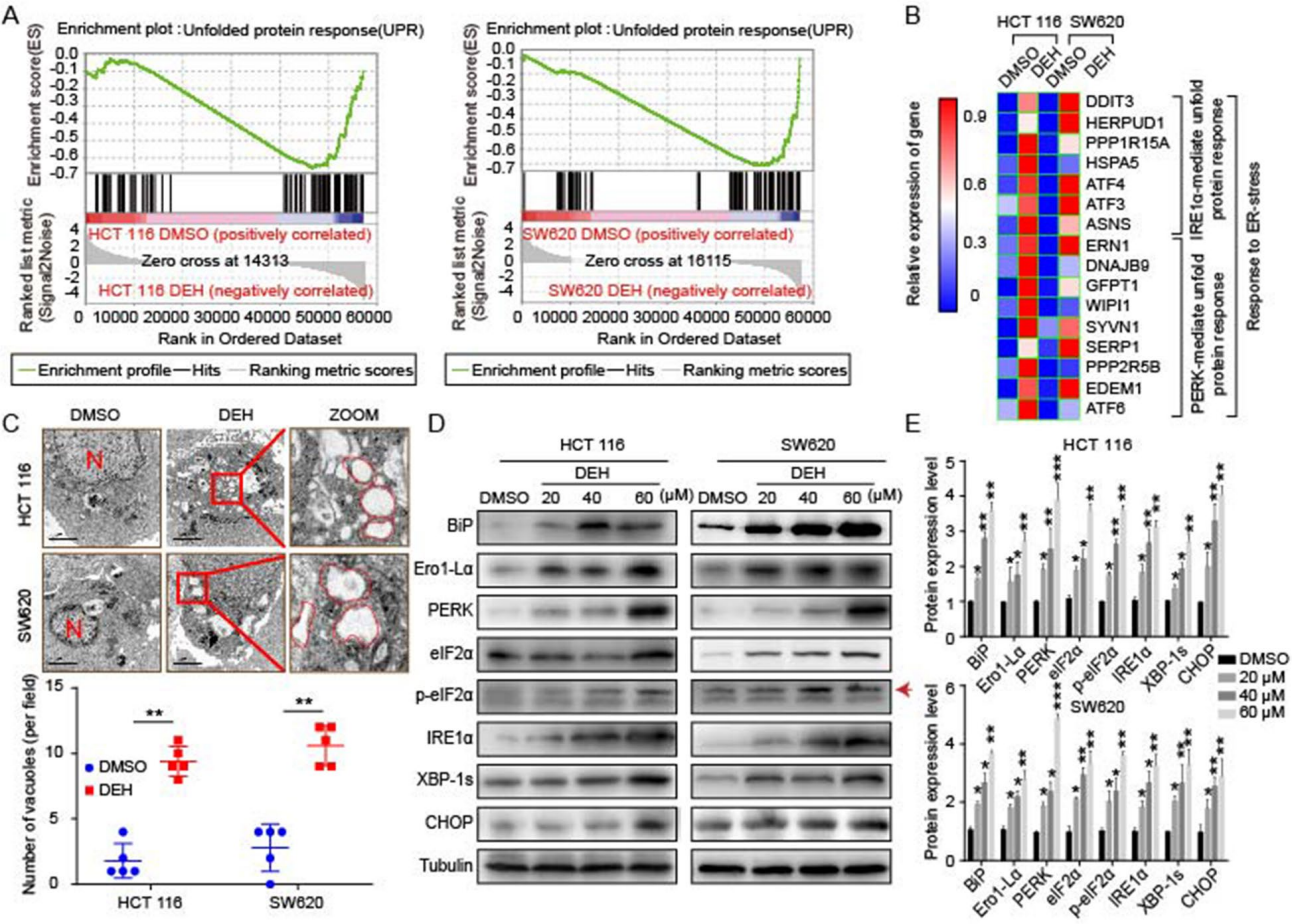
DEH induces ER stress in colorectal cancer cells. **a** Gene set enrichment analysis of UPR genes between control and DEH-treated cells. **b** The thermodynamic chart of the mRNA expression level of genes related to ER stress in colorectal cells after incubation with DEH for 48 h. **c** The subcellular structure of colorectal cancer cells after treatment with or without 60 μM DEH for 48 h were observed by TEM. Scale bar: 2 μm. N: nucleus. The ER is circled in red. **d** Western blotting assays were performed to detect the expression of BiP, Ero1-Lα, PERK, eIF2α, p-eIF2α, IRE1α, XBP-1 s, CHOP, and Tubulin in HCT 116 and SW620 cells after treatment with or without DEH. The densitometry of western blotting in the right panel. The data were presented as means ± SD. All the data were analyzed by the Unpaired Student’s t-test and *p*-values less than 0.05 were considered to be statistically significant. *p < 0.05, **p < 0.01, ***p < 0.001

**Fig. 6:**
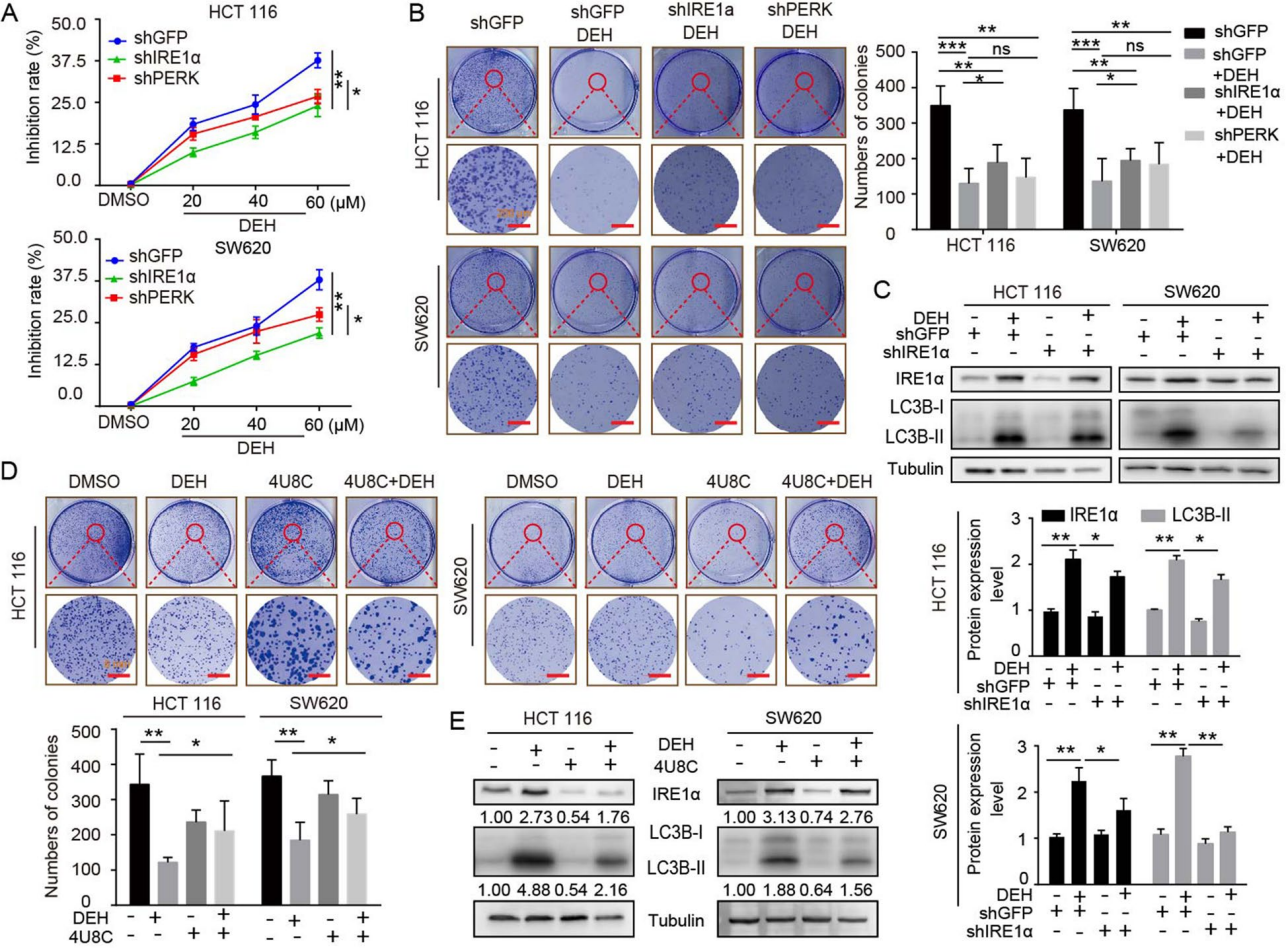
EDH induces autophagy through PERK/eIF2α and IRE1α/XBP-1 s/CHOP pathways in colorectal cancer cells. **a** The MTT assay was used to evaluate the inhibition rate of colorectal cells, which were transfected with PERK or IRE1α siRNAs, followed by incubation with the indicated concentrations of DEH for another 48 h. **b** The cell activity was detected by colony formation assay. Cells were transfected with PERK or IRE1α siRNAs, followed by incubation with 60 μM DEH for 10 days. The cells were stained with crystal violet staining solution. Scale bar: 200 nm. The number of clones was quantitated and presented to the right of the panel. **c** The western blotting assay was used to detect the expression of IRE1α, LC3B, and Tubulin. Tubulin was used as an internal control. **d** Cellular activity was also detected by colony formation assay. The cells were pretreated with 4U8C, followed by incubation with DEH for 10 days. The cells were stained with crystal violet staining solution. Scale bar: 200 nm. The number of clones were quantified and presented below the panel. **e** The expression of IRE1α, LC3B- II, and Tubulin was detected after DEH treatment with the inhibitor of IRE1α, 4U8C, or DMSO for 48 h. All the data were analyzed using the Unpaired Student’s t-test, and *p*-values less than 0.05 were considered to be statistically significant. *p < 0.05, **p < 0.01, ***p < 0.001

**Fig. 7:**
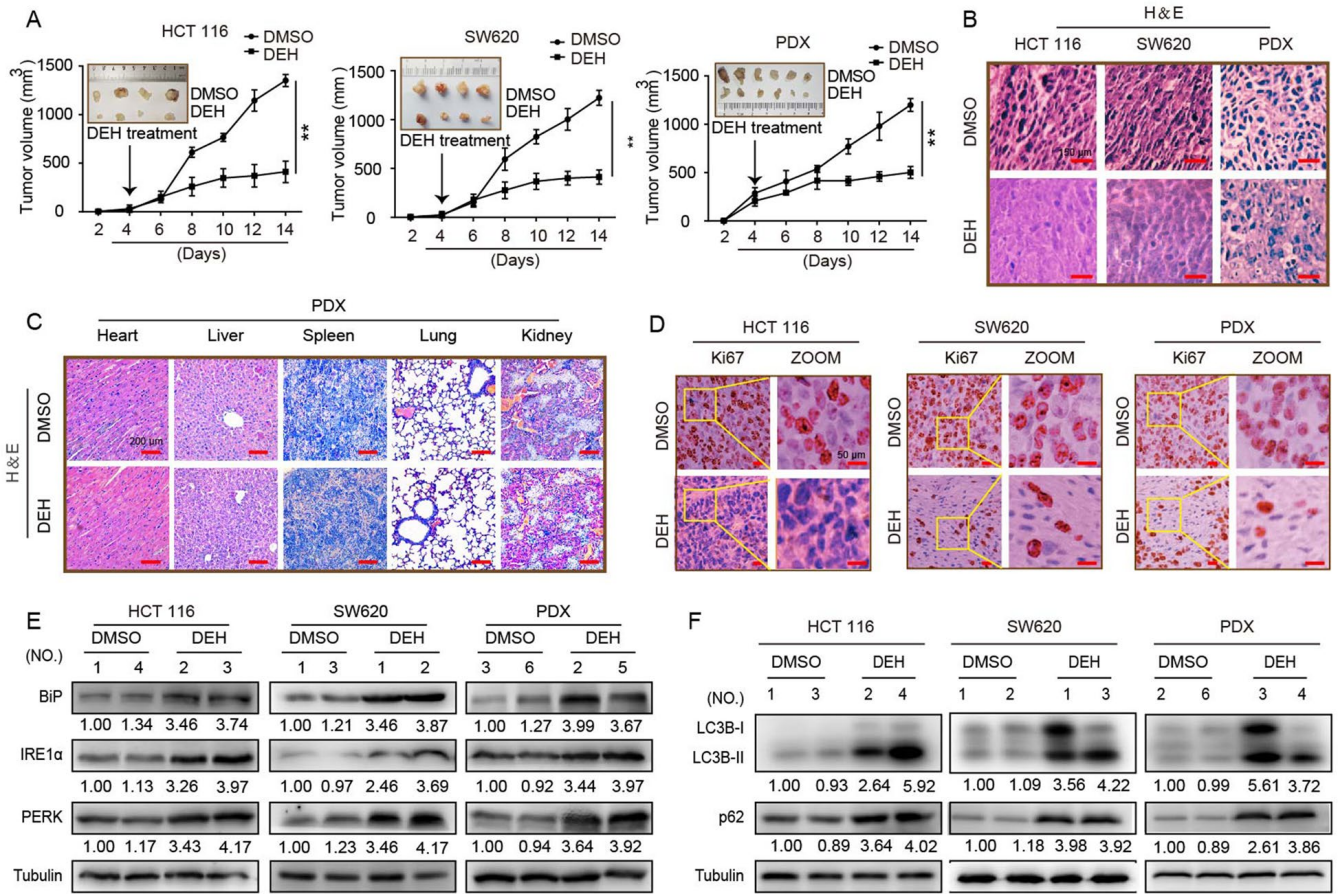
Effects of DEH on the growth of colorectal cancer in vivo. **a** HCT 116, SW620, and the tumor tissue from colon cancer patient were injected or transplanted into the flanks of NOD/SCID mice. The tumor-bearing mice were treated with DMSO or 40 mg/kg DEH by intraperitoneal injection when tumors were palpable. Tumor volume was measured every 2 days. Two weeks later, the mice were anesthetized and killed, and the tumors were imaged and analyzed. **b** Hematoxylin and eosin (H&E) staining of the indicated xenograft tumors. Scale bar: 150 μm. **c** H&E staining of the heart, liver, spleen, lung, and kidney in mice treated with DMSO or 50 mg/kg DEH. Scale bar: 500 μm. **d** Immunohistochemical (IHC) staining of the indicated xenograft tumors. Scale bar: 50 μm. **e** The expression of BiP, PERK, and IRE1α of xenograft tumors was detected by western blotting. **f** The expression of LC3B, p62, and tubulin in xenograft tumors was detected by western blotting. All the data were presented as means ± S.D. and are representative of three independent experiments. *P*-value < 0.05 was considered to be significant. **P < 0.01; ***P < 0.001
