# Supplementary figures and images for: Dehydrodiisoeugenol inhibits colorectal cancer growth by endoplasmic reticulum stress-induced autophagic pathways
Source: J Exp Clin Cancer Res. 2021 Apr 10;40:125. doi: 10.1186/s13046-021-01915-9 (PMC8035743; doi:10.1186/s13046-021-01915-9)

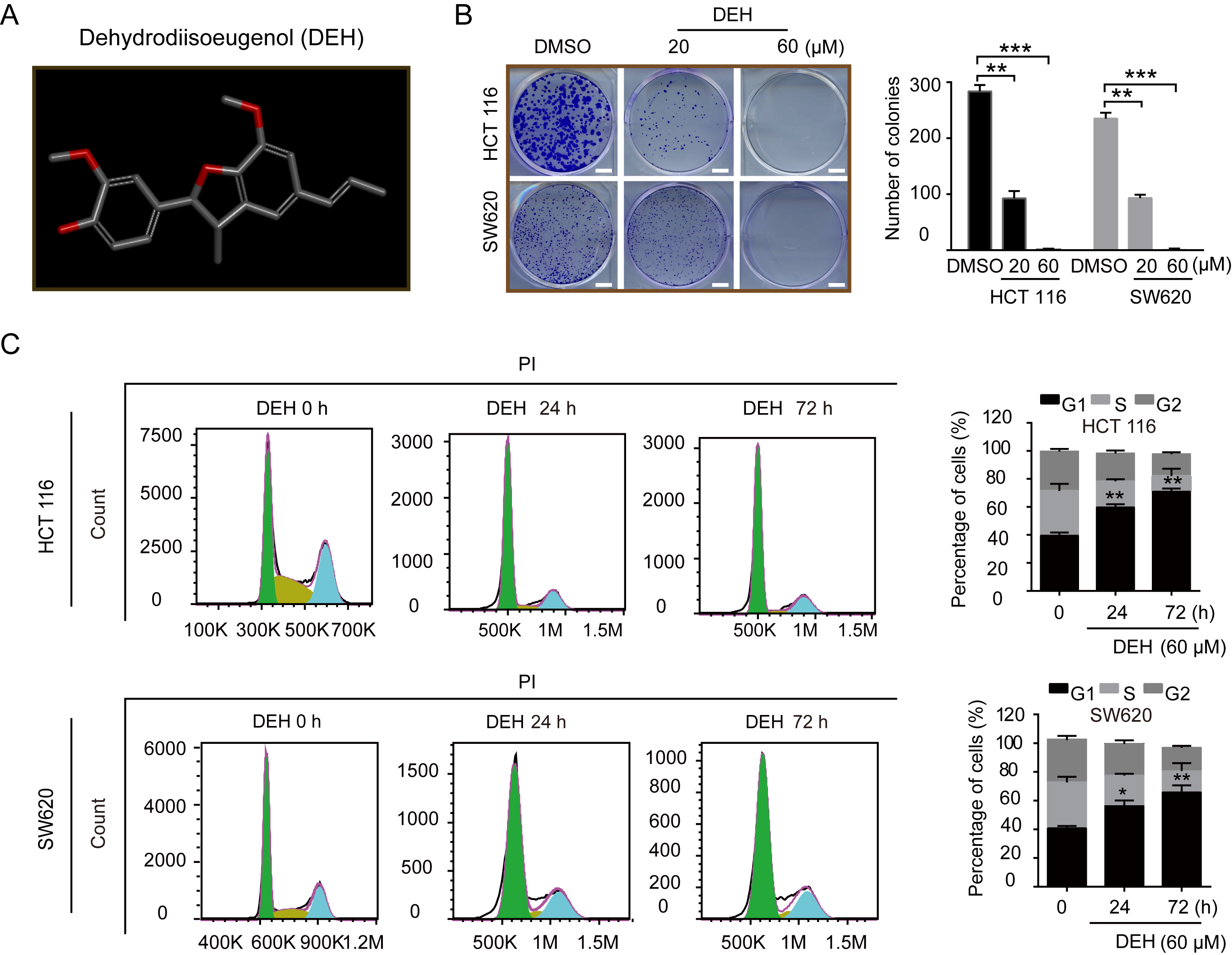

Supplement: Supplementary file 1 — Additional file 1: Figure S1. DEH inhibits cell growth of colorectal cancer cells, but not through apoptosis. A. The chemical structure of DEH used in this study. B. The plate colony formation assay was used to evaluate the cellular activity after treatment with DMSO or DEH. The numbers of clone were quantified and shown on the right of the panel. C. Cell cycles of HCT 116 and SW620 cells were investigated via flow cytometry after treatment with or without DEH for 24 h and 72 h. The distribution ratio of G1, S, and G2 of panel C was determined. All the data are means ±S.D. and are representative of three independent experiments. P-value less than 0.05 was considered to be statistically significant. ns: there was no significant difference, ***P < 0.001. [file 13046_2021_1915_MOESM1_ESM.tif]

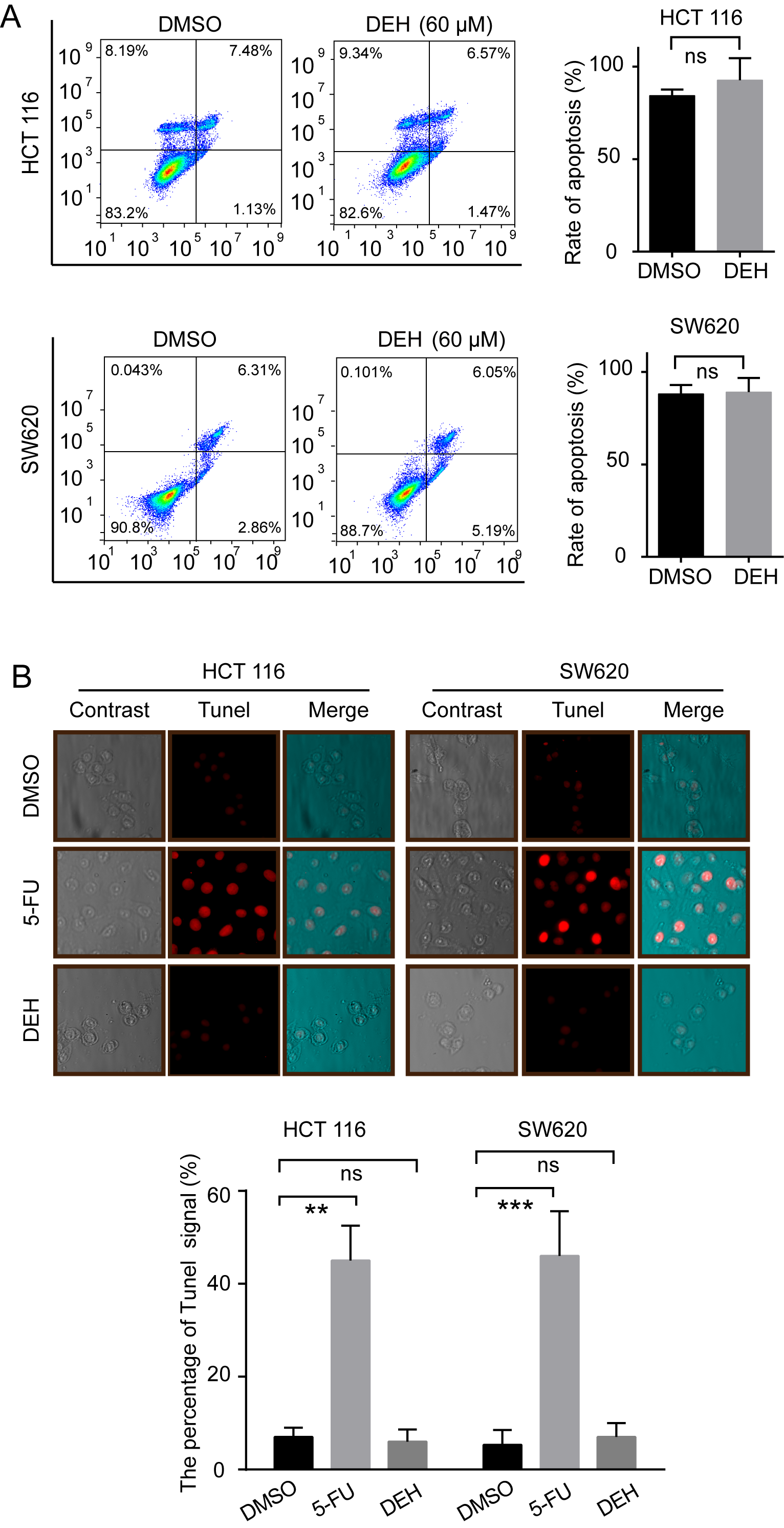

Supplement: Supplementary file 2 — Additional file 2: Figure S2. DEH could not induces apoptosis in colorectal cancer cells. A. HCT 116 and SW620 cells were incubated with DMSO or DEH for days, and the cell apoptosis was demonstrated by Annexin V/PI staining with flow cytometry. The cell apoptosis statistics were listed to the right of the panel. B. The immunostaining of TUNEL cell apoptosis detection was also used with DEH (60 μM), and the 5-FU (2 μM) was used as an positive control. The quantification was shown on the below of the panel. All the data are means ±S.D. and are representative of three independent experiments. P-value less than 0.05 was considered to be statistically significant. ns: there was no significant difference, ***P < 0.001. [file 13046_2021_1915_MOESM2_ESM.tif]

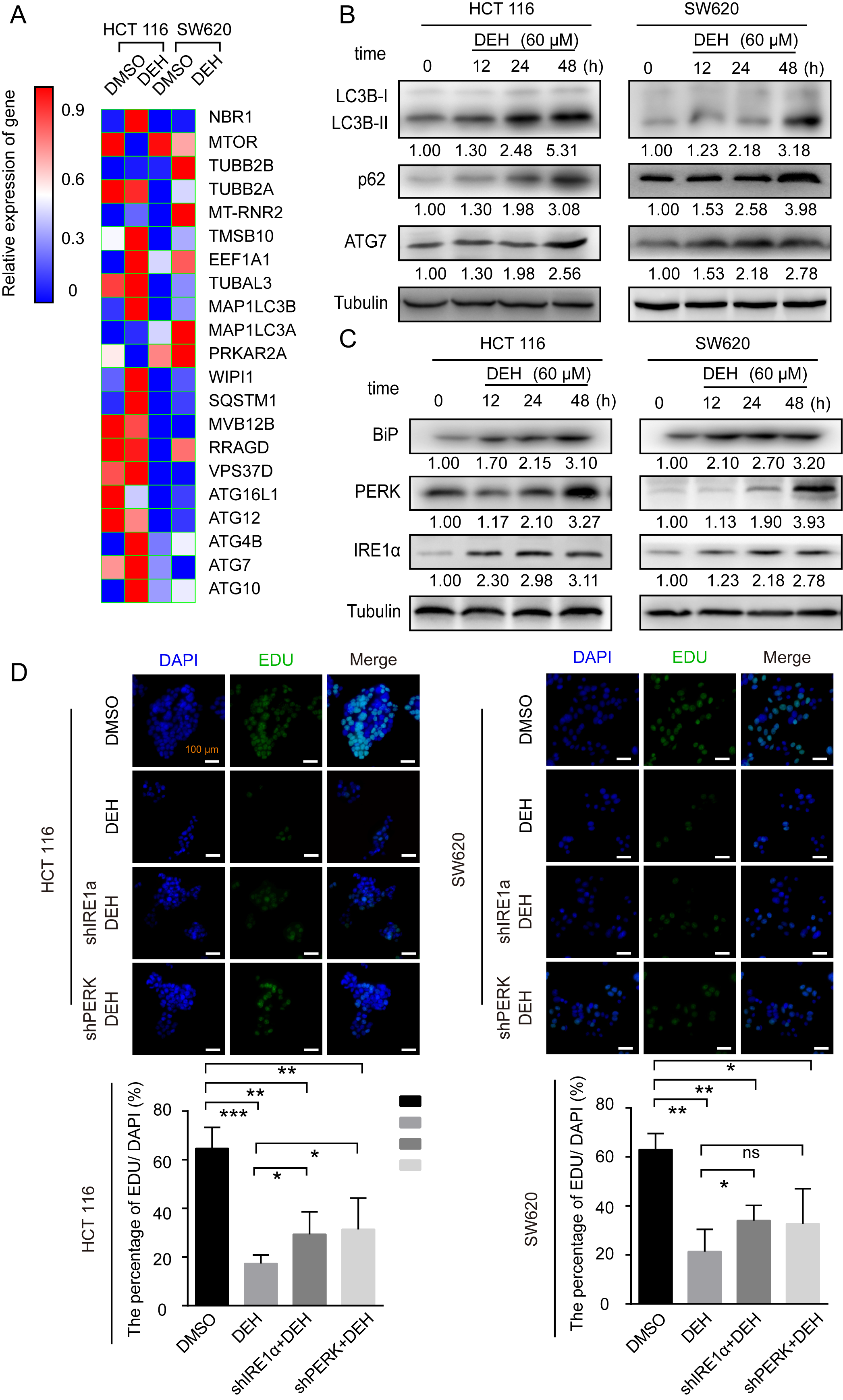

Supplement: Supplementary file 3 — Additional file 3: Figure S3. Expression profiles of autophagic and associated genes of colorectal cancer cells after treatment with DMSO or DEH. A. A heatmap of autophagic and associated genes of HCT 116 and SW620 cells after incubation with DMSO or DEH for 2 days. B. Western blotting was performed to investigate the expression of LC3B, p62, ATG7, and Tubulin in HCT 116 and SW620 cells after treatment with DEH. C. Western blotting was performed to investigate the expression of BiP, PERK, IRE1α, and Tubulin in HCT 116 and SW620 cells after treatment with DEH. Tubulin was used as a control. D. Images and quantification of -positive HCT 116 and SW620 cells after DEH treatment with PERK or IRE1α siRNAs for 48 h. Scale bar: 100 μm. Scale bar: 15 μm. The number of clones was counted and statistically represented as mean ± SD. The notability analysis was performed by the Unpaired Student’s t-test, and a p-value less than 0.05 was considered to be statistically significant. *p < 0.05, **p < 0.01, ***p < 0.001. [file 13046_2021_1915_MOESM3_ESM.tif]

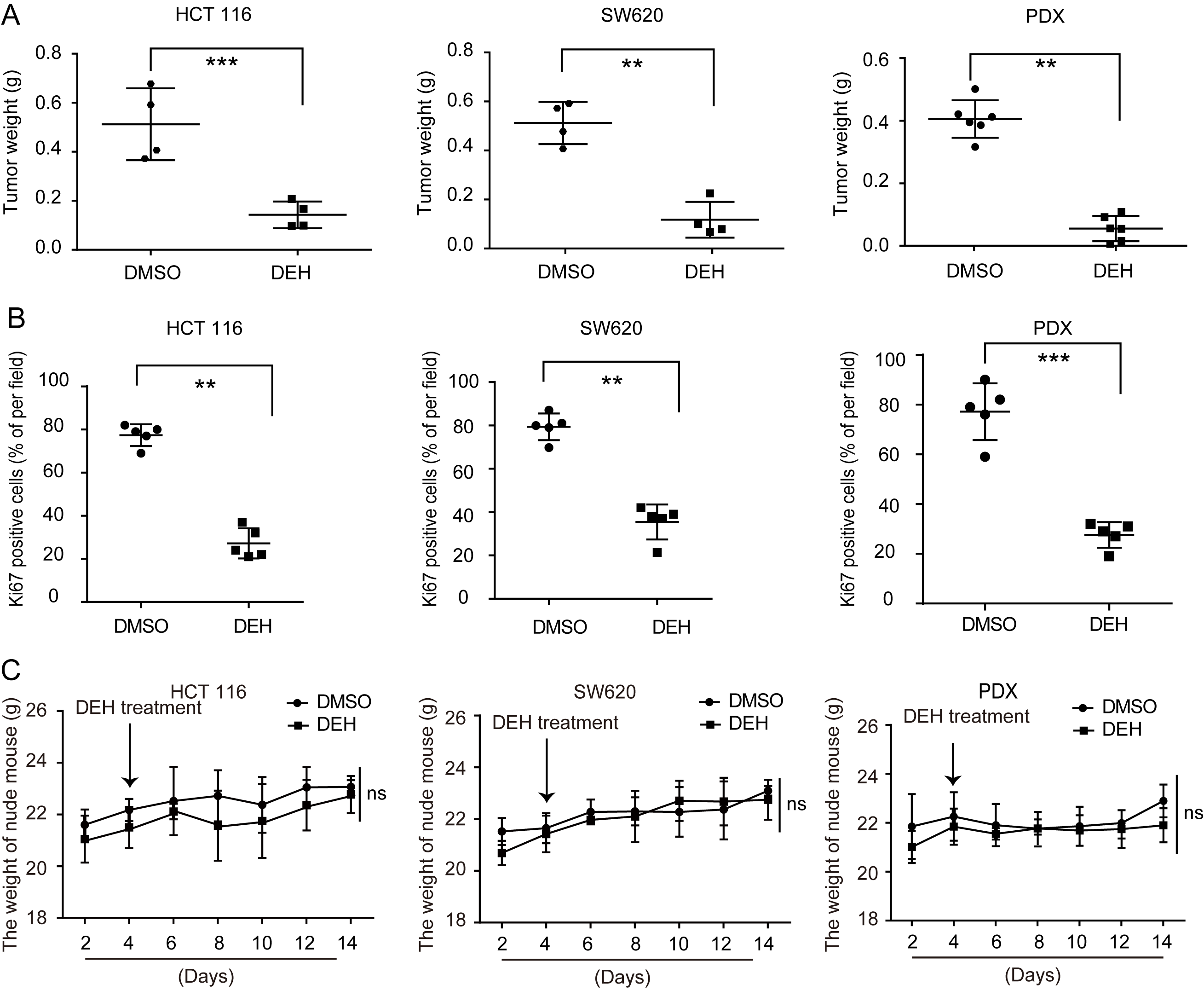

Supplement: Supplementary file 4 — Additional file 4: Figure S4. The anticancer activities of DEH on colorectal cancer were evaluated using CDX and PDX models in vivo. A. Quantified results of tumor weight. B. The Ki67-positive cells of tumor-bearing mice after treatment with DMSO or DEH were quantified and shown in the bar chart. C. The weights of the tumor-bearing mice were measured after treatment with DMSO or DEH for 2 weeks. All the data are presented as means ±S.D. and represent three independent experiments. P-value < 0.05 was considered to be significant. **P < 0.01; ***P < 0.001. [file 13046_2021_1915_MOESM4_ESM.tif]

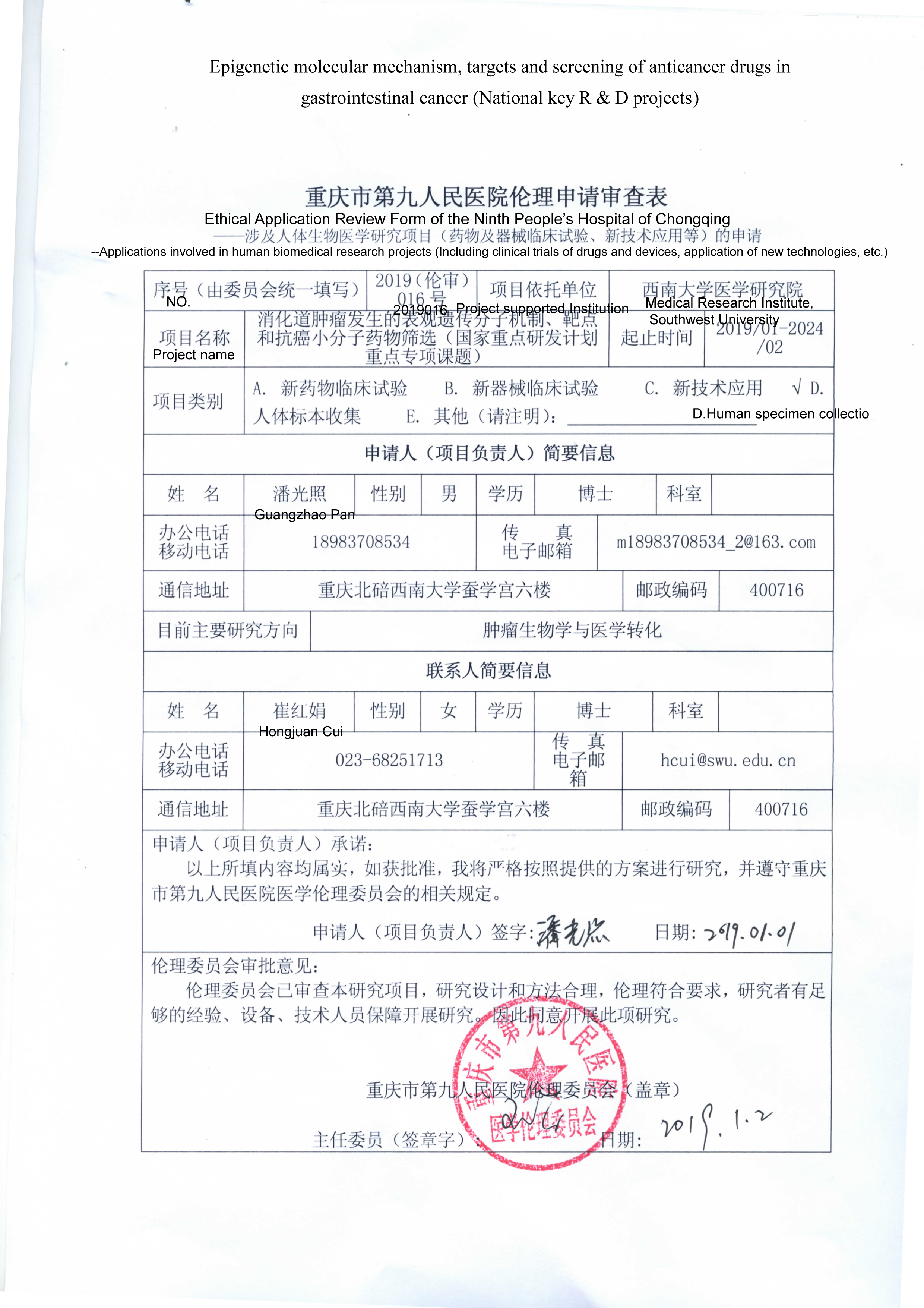

Supplement: Supplementary file 5 — Additional file 5: Figure S5. The ethics review. [file 13046_2021_1915_MOESM5_ESM.zip › The ethical review of gastrointestinal cancer_ESM.tif]

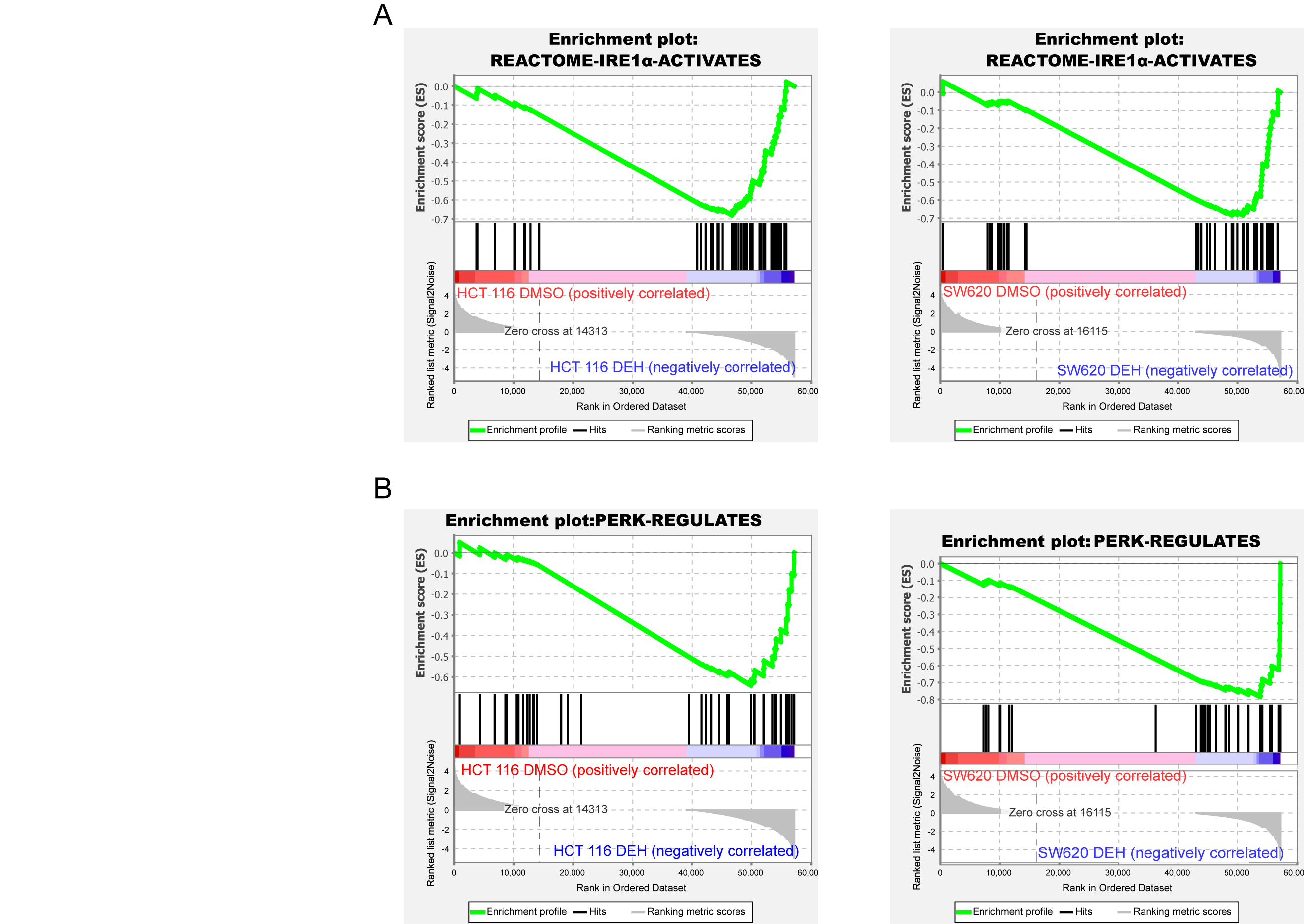

Supplement: Supplementary file 5 — Additional file 5: Figure S5. The ethics review. [file 13046_2021_1915_MOESM5_ESM.zip › The gene set enrichment analysis of ER stress_ESM.tif]
